# Exploring the patient experience of chronic hepatitis D (CHD) and assessment of content validity of the Hepatitis Quality of Life Questionnaire and (HQLQv2) and the Fatigue Severity Scale (FSS)

**DOI:** 10.1186/s41687-025-00903-3

**Published:** 2025-07-07

**Authors:** Pietro Lampertico, Aishwarya Chohan, Hannah Elwick, Nicola Williamson, Rowena Jones, Alon Yehoshua, Caroline Burk, Marvin Rock, Robert Gish, Nancy Reau, Heiner Wedemeyer, Maria Buti

**Affiliations:** 1 Division of Gastroenterology and Hepatology, Foundation IRCCS Ca’ Granda Ospedale MaggiorePoliclinico, Milan, Italy; 2 CRC “A.M. and A. Migliavacca” Center for Liver Disease, Department of Pathophysiology and Transplantation, University of Milan, Milan, Italy; 3 Patient-Centered Outcomes, Adelphi Values Ltd., Bollington, United Kingdom; 4 Gilead Sciences, Forester City, California USA; 5 Hepatitis B Foundation, Doylestown, PA USA; 6 Department of Internal Medicine, Division of Digestive Diseases and Nutrition, Rush Medical College, Chicago, USA; 7 Department of Gastroenterology, Hepatology, and Endocrinology, Hannover Medical School, Hannover, Germany; 8 Liver Unit, Hospital Universitario Valle Hebron and Ciber-ehd del Instituto Carlos III, Barcelona, Spain

**Keywords:** Cognitive debriefing (CD), Concept elicitation (CE), Content validity, Fatigue severity scale (FSS), Health-related quality of life (HRQoL), Hepatitis D virus (HDV), Hepatitis quality of life questionnaire (HQLQ), Patient experience, Patient-reported outcome (PRO), Qualitative interviews

## Abstract

**Background:**

Chronic hepatitis D (CHD) is the most severe form of viral hepatitis, which results in accelerated progression to cirrhosis and poor prognosis compared with other hepatitis infections, impacting patients’ health-related quality of life (HRQoL). To adequately capture patient perspectives of new hepatitis D virus (HDV) treatments in clinical trials, patient-reported outcome (PRO) measures that are valid and assess key concepts relevant to the patient are needed. This study aimed to explore the patient experience of CHD and evaluate the content validity of the Hepatitis Quality of Life Questionnaire (HQLQv2) and the Fatigue Severity Scale (FSS) for use in an HDV population.

**Methods:**

Combined qualitative concept elicitation (CE) and cognitive debriefing (CD) interviews were conducted with 39 patients in Germany, Italy, Spain, and the US with a clinician-confirmed diagnosis of CHD. Participants described their experience of CHD, informing the development of a conceptual model, and then completed the HQLQv2 and FSS using a think-aloud technique to assess understanding, relevance, and comprehensiveness of items, instructions, response scales, and recall periods. Interviews were conducted in the principal language of each country; official translations of the instruments were used, and all patient-facing study documents and the interview guide were translated by certified translators.

**Results:**

The sample included participants with a range of liver fibrosis stages, including 11 with compensated (*n* = 9) and decompensated (*n* = 2) cirrhosis. Fatigue, loss of appetite, nausea, joint pain, and pain over the liver were the most frequently reported signs/symptoms. Fatigue was most commonly mentioned and was described as a severe and particularly burdensome symptom, that impacted several aspects of patients’ daily lives. Participants reported that CHD impacted their emotional wellbeing (low mood, anxiety), physical functioning (difficulty walking), social functioning (attending social events), activities of daily living (household chores), and work. Participants demonstrated a good understanding of the HQLQv2 and FSS items, instructions, response scales and recall periods, and the concepts assessed were considered relevant to CHD by most participants.

**Conclusion:**

Findings contribute to the understanding of the patient experience of CHD and support content validity of the HQLQv2 and FSS as outcome assessments for use in an HDV population.

**Supplementary Information:**

The online version contains supplementary material available at 10.1186/s41687-025-00903-3.

## Introduction

Chronic hepatitis D (CHD) is the most severe form of viral hepatitis in humans and requires co-infection with hepatitis B virus (HBV) for its replication. Approximately 4.5% of patients who have chronic hepatitis B (CHB) are estimated to be infected with hepatitis D virus (HDV), corresponding to an estimated 12 million people globally [1]. Patients with CHD may experience accelerated progression to cirrhosis and poorer prognosis compared with other viral hepatitis infections. Relative to HBV, HDV carries a three-fold risk for the development of hepatocellular carcinoma (HCC), and a two-fold risk for developing decompensated liver disease and mortality [2]. Disease progression to cirrhosis occurs rapidly, affecting between 70.0 and 80.0% of patients within 5–10 years and at a younger age [3, 4].

The symptoms of HDV are indistinguishable from other forms of viral hepatitis and include fatigue, loss of appetite, nausea, vomiting, joint pain, and abdominal pain [5, 6]. No qualitative studies appear to exist that explore the signs/symptoms and health-related quality of life (HRQoL) impacts of patients with HDV; however, a recent qualitative study in CHB found that patients experience significant emotional and psychological impacts, which affect their lifestyles, relationships, and work/schooling [7]. Previous quantitative research suggests that while HRQoL is impacted in both CHD and CHB patients, CHD causes more significant impacts on aspects of physical functioning, emotional wellbeing, work productivity, and daily activities than CHB [8]. The absence of qualitative research exploring the patient experience of CHD highlights the need for in-depth studies in this patient population. Detailed patient insights can be obtained through qualitative research to enable the identification of important and relevant concepts [9, 10] and can inform the selection of suitable patient-reported outcome (PRO) measures for use in HDV clinical trials assessing the efficacy of new treatments [9–11] and support generation of value propositions for new products [12].

HDV is most commonly treated off-label using interferon alpha (IFNα) or pegylated IFNα (peg- IFNα) [13]. These therapies, however, have been shown to negatively affect patients’ HRQoL [14] and are associated with poor response rates and limited efficacy, with only 10.0-20.0% of patients achieving sustained HDV clearance following 1-year course of treatment [15, 16]. Additionally, due to contraindications, IFNα treatments are not suitable for patients with autoimmune diseases, advanced or decompensated liver disease, and the elderly [17]. Bulevirtide is a first-in-class anti-viral that has shown promise for the treatment of CHD in adults [18, 19]. In a phase 2 clinical trial enrolling patients with CHD, bulevirtide monotherapy was shown to induce a dose-dependent reduction in HDV RNA and improvements in liver disease, with undetectable HDV RNA levels observed in ≥ 50.0% of patients treated with bulevirtide + tenofovir compared to tenofovir alone [20]. In the phase 3 clinical trials, 12.0% and 51.0% of patients who received 2 mg and 10 mg bulevirtide monotherapy daily had undetectable HDV RNA levels at Week 48, compared with only 2% of patients who received no treatment for 48 weeks [21].

To evaluate the potential effect of bulevirtide on patients’ HRQoL, the Hepatitis Quality of Life Questionnaire version 2 (HQLQv2) and the Fatigue Severity Scale (FSS) were included as exploratory endpoints in the bulevirtide phase 3 clinical trials. These instruments were selected as they have been validated for use in related conditions (e.g., hepatitis B and C) and no disease-specific PRO measures currently exist for assessment of HRQoL in CHD [22–24]. The HQLQv2 is a 51-item PRO measure developed to assess the functional health and wellbeing of patients with hepatitis C virus (HCV) and comprises the Short Form-36 (SF-36; a generic measure of a patient’s health status) and four hepatitis-specific domains (i.e., health distress, positive wellbeing, hepatitis-specific limitations, and hepatitis-specific health distress) [25]. Improvements in the domains of hepatitis-specific limitations and hepatitis-specific health distress are the main focus for assessment of HRQoL in the phase 3 trial. The FSS comprises nine items designed to assess the severity of fatigue on daily activities and lifestyles across health conditions [26]. While both PROs assess HRQoL concepts that are likely relevant to the patient experience of HDV, content validity of these measures in an HDV population has not yet been established.

Global best practice guidance for patient-focused drug development specifies that content validity should be assessed using qualitative methods to generate evidence that the content of a PRO is relevant, comprehensive, and comprehensible within the target population and for the specific context of use. For PROs intended to serve as clinical endpoints (including those considered exploratory) to evaluate treatment benefit, this includes an evaluation of how well the content and structure of a PRO aligns with its intended measurement concept and the extent to which the concepts assessed by a PRO are clinically relevant and important to how patients ‘feel and function’ [9, 11, 27–29].

The objective of this research was to conduct qualitative research with adults with a confirmed diagnosis of CHD to explore the patient experience, including relevant signs, symptoms and HRQoL impacts, and evaluate content validity of the HQLQv2 and FSS for their suitability for use in an HDV population.

## Methods

### Study design

**Fig. 1:**
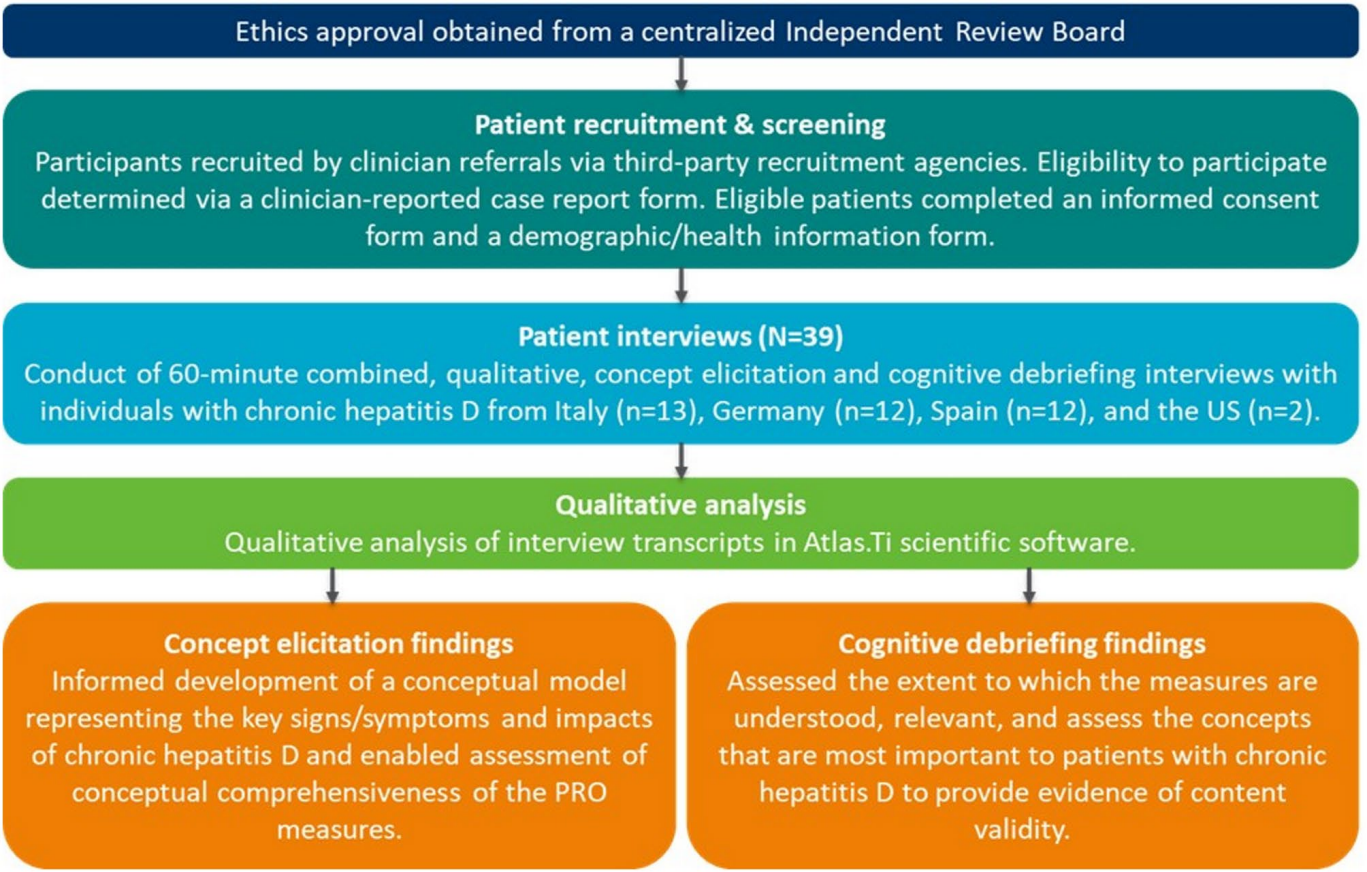
Overview of study design

This was a non-interventional, cross-sectional, qualitative interview study comprising combined concept elicitation (CE) and cognitive debriefing (CD) interviews with adults with CHD from Germany, Italy, Spain, and the US. CE explored the patient experience of CHD, informing the development of a conceptual model to support assessment of conceptual comprehensiveness of the HQLQv2 and FSS. CD assessed whether the HQLQv2 and FSS are understood, relevant, and capture all concepts important to patients. All participant-facing study documents and the interview guide were translated by certified translators prior to the conduct of any interviews. All translators were fluent in English and the target language and held a bachelor’s degree in translation and interpretation at minimum. Official translations of the HQLQv2 and FSS in German, Italian, and Spanish were also obtained from the licensees for use during the CD portion of the interviews. Figure 1 provides an overview of the study design.

### Sample and recruitment

Patients were recruited by specialist recruitment agencies via referring clinicians in Italy, Germany, Spain, and the US. Eligible patients had to be at least 18 years of age, have a clinician-confirmed diagnosis of CHD for at least six months following a positive polymerase chain reaction (PCR) test result for HDV Ribonucleic Acid (RNA) as well as a confirmed liver disease stage of F0-F3 or F4 (compensated or decompensated cirrhosis) and/or HCC. Patients were excluded if they had an acute episode of liver disease during the last 6 months (non-cirrhotic: acute hepatic injury; cirrhotic: new onset hepatic encephalopathy (HE), ascites, variceal bleeding), had been treated with IFN/peg-IFN during the last 6 months, had ever received bulevirtide for the treatment of HDV or HBV, were heavily immunocompromised, had been diagnosed with human immunodeficiency virus (HIV), had ever received any organ transplant(s), or had been diagnosed with HCV.

The target sample size was driven by the principles of concept saturation (i.e., the point at which no new concept-relevant information is likely to emerge with further interviews) [30]. Based on previous research, a minimum of 12 patients in total was deemed sufficient [31]. Given the rarity of chronic HDV infection, patients were recruited from three countries in Europe and the US to maximize recruitment. However, no differences in the patient experience were expected between countries. Target sampling quotas were also set to encourage representation of patients with a range of clinical and demographic characteristics within the sample (e.g., age, liver fibrosis stage, time since diagnosis). All participants were compensated for their participation.

### Interview procedure

The study was approved and overseen by Western Copernicus Group Independent Review Board (WCG IRB; IRB tracking numbers: 45145622, 20222536). All participants provided written informed consent prior to commencing any study-related activities. Interviews were 60 min and conducted via telephone by six experienced qualitative interviewers from Adelphi Values Patient-Centered Outcomes (US participants; *n* = 2 interviewers), Global Perspectives (German and Italian participants; *n* = 2 interviewers), and Pharmore (Spanish participants; *n* = 2 interviewers) in the principal language of the target country, using a semi-structured interview guide (Supplementary File S1). All interviewers were briefed on the interview guide and the objectives of the study prior to conducting any interviews.

The CE section of the interviews was exploratory and focused on eliciting information regarding the patient experience of signs, symptoms, and impacts of CHD. Interviews began with broad, open-ended (e.g., “*Please tell me about your current experience of HDV? What is it like to live with HDV?”*) questions to facilitate spontaneous elicitation. Open-ended questioning was followed by more focused questions, designed to probe participants on topics of interest that they may not have mentioned during the interview or that required further elaboration or clarification (e.g., *“How would you rate fatigue on a typical day on a scale of 0–10*,* where 0 is not at all severe and 10 is the worst you can imagine? Can you tell me why you chose that rating”*). Participants were also asked which symptoms were most bothersome and which were most important to treat.

For the CD section, participants were asked to complete the HQLQv2 and FSS on paper using a ‘think aloud’ approach [32], where they were asked to share their thoughts as they read each instruction and item and selected each response. Participants were then asked detailed questions about their interpretation and understanding of instruction and item wording, relevance of concepts, and appropriateness of response options and recall periods. General feedback on the questionnaires was also explored.

### Qualitative analysis

All interviews were audio-recorded, transcribed verbatim, and translated to English (by certified translators), with identifiable information redacted. The CE section of the transcripts were subject to thematic analysis [33, 34] using ATLAS.ti scientific software (Version 22) [35]. Participant quotes pertaining to the signs, symptoms and impacts of CHD were assigned corresponding concept codes in accordance with an agreed coding frame. Codes were applied both deductively (based on prior knowledge) and inductively (as emerging from the data). Coding of all translated transcripts was conducted by four investigators who were trained to use the coding frame and were familiar with the objectives of the research. The research team met regularly to identify and address any coding discrepancies and to check the accuracy of the coding across transcripts. Coding was reviewed and overseen by the project lead to further ensure consistency and quality. Analysis was conducted for the full sample and not for individual countries given the small sample size in each country.

Based on the CE findings, a conceptual model was developed in alignment with US FDA Patient-Focused Drug Development (PFDD) guidance [11] to display the key signs, symptoms, and impacts associated with CHD. The conceptual model was then used to assess conceptual comprehensiveness of the HQLQv2 and FSS. Concepts identified in the CE interviews were mapped onto the HQLQv2 and FSS to assess their conceptual coverage and to further determine the extent to which both instruments assess concepts relevant to the patient experience of CHD. As both the HQLQv2 and FSS are measures of HRQoL, the coverage of symptom concepts was not explored.

Saturation analysis was conducted on the CE interview data to determine the appropriateness of the sample size. Transcripts were chronologically grouped into four sets and spontaneously reported signs, symptoms, and impact concepts identified within each set were iteratively compared. Saturation was deemed to be achieved if no new symptom and impact concepts were identified in the final set of interviews [30].

The CD section of the interviews was analyzed using dichotomous codes that were assigned to each instruction, item, response option, and recall period to indicate whether it was understood, relevant, appropriate, and why. Further codes captured ease of completing and relevance of the questionnaires to the patient experience of CHD.

## Results

### Sample characteristics

A total of 39 adults with CHD from Italy (*n* = 13), Germany (*n* = 12), Spain (*n* = 12), and the US (*n* = 2) were interviewed as part of the study. Participants ranged in age from 20 to 73 years (median: 55 years) and most were female (64.1%), had a liver fibrosis stage of F0-F1 (51.3%), and had received their HDV diagnosis more than 4 years prior to the interview (59.0%). Table 1 provides an overview of the sample characteristics alongside the corresponding target quotas.

**Table 1:** Participant demographic and clinical characteristics and achievement of target recruitment quotas (*N* = 39)

Criteria	Target, *N*	Actual, *N* (%)
**Sex**		
Male	≥ 12	14 (35.9%)
Female	≥ 10	25 (64.1%)
**Age**		
18–30	≥ 9	3 (7.7%)
31–55	≥ 14	17 (43.6%)
> 55	≥ 6	19 (48.7%)
**Education level**		
Completed high school or below	≥ 12	18 (46.2%)
Completed some college/degree or above	≥ 12	21 (53.8%)
**Liver fibrosis stage**		
F0-F1	≥ 6	20 (51.3%)
F2	≥ 5	6 (15.4%)
F3	≥ 5	2 (5.1%)
F4 (compensated cirrhosis)	≥ 4	9 (23.1%)
F4 (decompensated cirrhosis)	≥ 4	2 (5.1%)
**Diagnosis of HCC**		
Not diagnosed with HCC	≥ 10	37 (94.9%)
Diagnosed with active HCC	≥ 6	2 (5.1%)
**Time since CHD diagnosis**		
1 month-1 year	≥ 6	4 (10.3%)
1 year-4 years	≥ 6	11 (28.2%)
> 4 years	≥ 6	23 (59.0%)
HBV treatment status*		
Untreated	No target	2 (5.1%)
Previously treated	No target	11 (28.2%)
Currently treated	No target	26 (66.7%)

*Per the exclusion criteria, no patients were included in this study if they had ever received bulevirtide for the treatment of CHD or CHB or if they had received INF/peg-INF within six months prior to the interview

Target sampling quotas were achieved for sex, age (≥ 31 years), education level, liver fibrosis stage (F0-F1, F2, F4 – compensated), diagnosis of HCC (not diagnosed), and time since diagnosis (≥ 1 year), but missed for age (18–30 years), liver fibrosis stage (F3, F4 – decompensated), diagnosis of HCC (diagnosed), and time since diagnosis (1 month-1 year) due to recruitment challenges.

### Concept elicitation

**Fig. 2:**
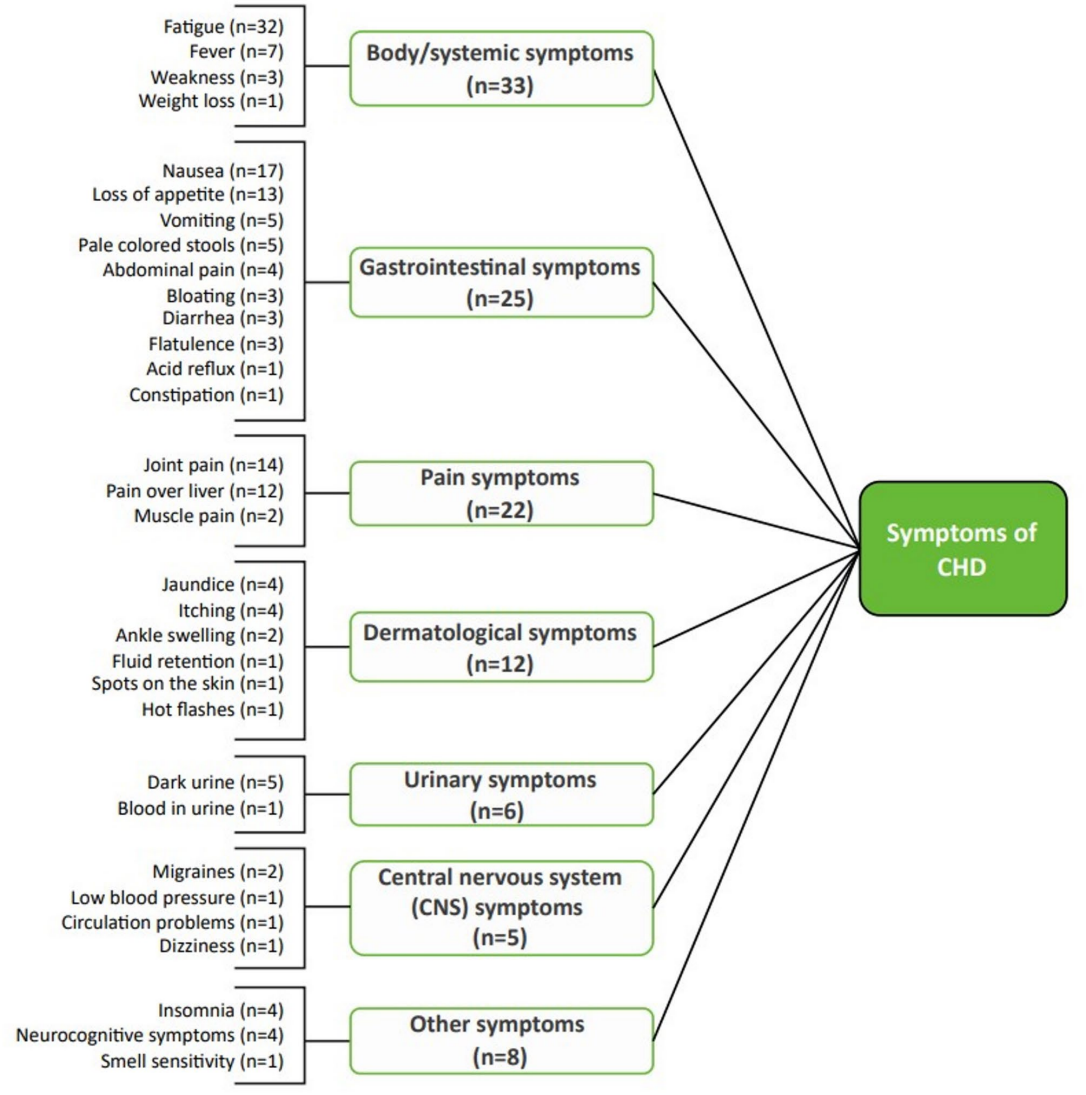
Conceptual model of CHD – Symptoms (*n* = 39)

**Fig. 3:**
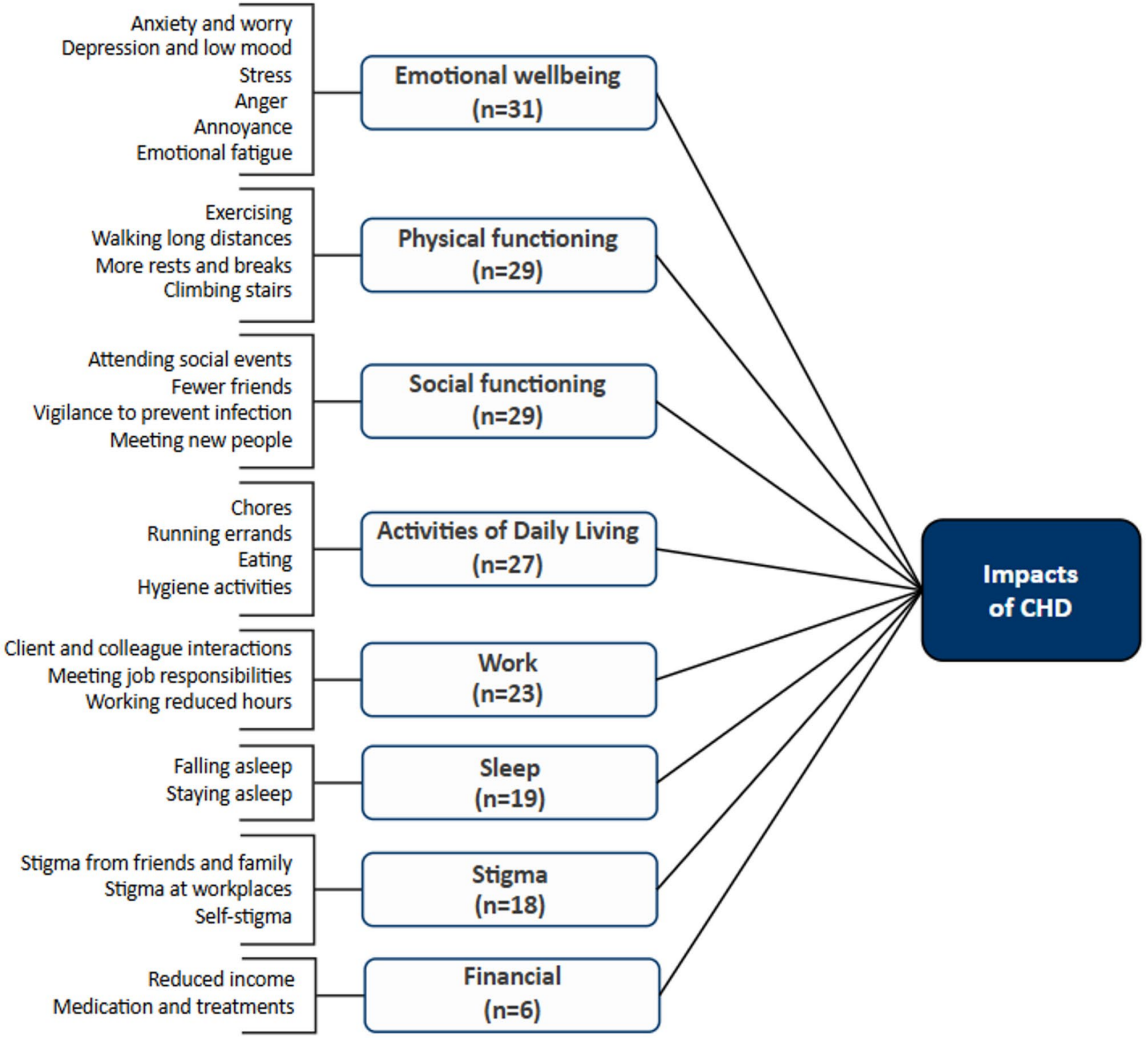
Conceptual model of CHD – Impacts (*n* = 39)

The findings from the CE portion of the interviews are summarized in a conceptual model, displaying the key signs and symptoms (Fig. 2) and impacts (Fig. 3) associated with CHD.

### Signs and symptoms

As shown in the conceptual model (Fig. 2), participants reported a total of 32 signs and symptoms during the interviews, broadly categorized as four body/systemic symptoms, ten gastrointestinal symptoms, three pain symptoms, two urinary symptoms, six dermatological symptoms, four central nervous symptoms, and three other symptoms. Body/systemic symptoms (specifically fatigue), gastrointestinal symptoms (specifically nausea and loss of appetite), and pain symptoms (specifically joint pain and pain over liver) were most commonly mentioned. Exemplary participant quotes for the five most mentioned symptoms are provided in Table 2. Generally, similar proportions of participants with each stage of liver fibrosis reported experiencing the five most frequently reported signs/symptoms during the interviews.

**Table 2:** Overview of most frequently mentioned signs and symptoms during the interviews (*N* = 39)

Sign/Symptom, *n* (%)	Symptom rated most bothersome	Symptom severity rated between 7 and 10*	Example supportive quotes
Fatigue (*n* = 32, 82.1%)	14/22 (63.6%)	19/32 (59.4%)	*“It became really clear that I’m frequently tired and run down. Or else*,* when I perform strenuous tasks*,* I’m fatigued sooner than previously.”* (Participant 6)
Nausea (*n* = 17, 43.6%)	1/22 (4.6%)	4/11 (36.4%)	*“I also have nausea even though I don’t vomit […] Every day. I just woke up from my nap and I’m feeling this nausea right now.”* (Participant 33)
Joint pain (*n* = 14, 35.9%)	2/22 (9.1%)	7/15 (46.7%)	“*I experience joint pain in the whole body*,* that means… in the legs as well as in the arms. […] with the outbreak of the disease there were few symptoms but it’s getting worse year after year.”* (Participant 38)
Loss of appetite (*n* = 13, 33.3%)	Not reported	2/9 (22.2%)	“*I don’t know how to say it*,* a sense of lack of appetite*,* when you don’t want to eat or see food or in the morning when you don’t want to have breakfast. This is what happens to me*.” (Participant 24)
Pain over liver (*n* = 12, 30.8%)	Not reported	4/9 (44.4%)	*“Well a little bit*,* but actually [there’s] a pressure on my liver. Such a pressure in the upper stomach area*,* on the right sight. A little bit of that. That’s something I experience sometimes.”* (Participant 9)

*Participants were asked to rate the symptom on a typical day from 0 = not at all severe to 10 = as severe as you can imagine

Most notably, fatigue was discussed by the vast majority of participants (*n* = 32/39; 82.1%) and was commonly described as severe (*n* = 19/39; 48.7%) and the most bothersome sign/symptom (*n* = 14/22; 63.6%). Most participants experienced fatigue either daily (*n* = 10/32; 31.3%) or weekly (*n* = 12/32; 37.5%) and referred to it as being ‘tired’, ‘run down’, ‘wiped out’, or having ‘no energy’. Nearly all participants who were asked noted their fatigue had worsened over time (*n* = 8/11; 72.7%).

### HRQoL impacts of CHD

The impacts of CHD are grouped into eight domains, as shown in the conceptual model (Fig. 3): emotional wellbeing, social functioning, physical functioning, activities of daily living (ADL), work, sleep, stigma, and finances (see Table 3 for example quotes). Participants mostly discussed impacts spontaneously (without interview probing) and the proportion of participants with each stage of liver fibrosis reporting on the various domains was fairly consistent.

**Table 3:** Overview of impact domains reported by participants during the interviews (*N* = 39)

Impact domain	Number of participants (*N* = 39)	Example supportive quotes
Emotional wellbeing	*n* = 31/39	*“Now the most relevant impact is at emotional level*,* because everything was well controlled and about a year and a half*,* the blood test showed some altered values and it’s ever since then that I started to get worried.”* (Participant 14) *“Living with nausea makes me feel depressed. You always need to be thinking what you can eat or drink to alleviate it.”* (Participant 33)
Social functioning	*n* = 29/39	*“Look*,* I used to go out before. I used to go out at night or I used to go to the local feasts… I haven’t gone this past summer because I said*,* “I won’t be able to stand it”. As soon as I start dancing the first ten dances*,* I need to go back home. Or*,* if my friends ask me to go dancing*,* I directly answer no.”* (Participant 15) *“Well*,* it affects me in the sense*,* I used to be obsessed with the idea of infecting someone… I still watch out with these things*,* when they say “taste this” and the other one says “I also want to taste it” I say “ok*,* you taste it first and I’ll taste it when you finish”*,* and things like that. And the same with anyone else.”* (Participant 16)
Physical functioning	*n* = 29/39	*“It limits what I have to do: going to the gym. I reduced the time I spent going to the gym. I go to the gym but I have to keep it in mind that I have to limit myself because I can’t do everything.”* (Participant 28) *“I cannot do long walks of 20 km because after 4-5km I feel tired*,* and I need to stop and rest.”* (Participant 21)
Activities of daily living	*n* = 27/39	“…*I find it hard to clean the flat. Yes*,* yes*,* that’s a big limitation. Me…I find it difficult to get dressed… That means*,* if I can’t get my arms up*,* of course I have a problem getting dressed*,* as well as blow-drying my hair*,* washing my hair… I don’t dare to take a bath anymore*,* I only take showers*,* it doesn’t work at all…”* (Participant 38) “*After half an hour of vacuum-cleaning I am completely exhausted. I have the feeling that I have carried 100 bottles to the basement.”* (Participant 5)
Work	*n* = 23/39	*“I have to do everything at a slower pace. I can’t perform so well. I can work and all that. But I need to do it calmly*,* slowly and in a focused manner. I can tell that it has changed in reference to the past.”* (Participant 39) *“When I used to work*,* I did have exhausting shifts*,* from 16 to 4 in the morning*,* then I was always tired.”* (Participant 13)
Sleep	*n* = 19/39	*“It happened that nausea woke me up from my sleep. I was sleeping and suddenly*,* I don’t know why.”* (Participant 20)
Stigma	*n* = 18/39	*“Well*,* they [friends and family] tend to isolate me because they are afraid… they see you through different eyes in comparison to the past [before disclosing HDV diagnosis]”* (Participant 34) *“They got to ask me whether I had been a drug addict. They asked me not to get close to their son…”* (Participant 15)
Finances	*n* = 6/39	*“I have to make co-payments for my medication. And of course*,* I can’t work as much. That’s why I have less income now.”* (Participant 39) *“Especially here where health treatments are very costly*,* even if you have insurance.”* (Participant 22)

Impacts on emotional wellbeing were most frequently mentioned and included anger and annoyance at receiving an HDV diagnosis and anxiety or worry about transmission and disease progression. Participants also described feeling depressed or sad about their reduced quality of life and inability to perform activities in the same way as healthier individuals, as well as feelings of stress and general emotional fatigue due to a lack of energy and poor health. In terms of social functioning, participants reported the loss of friendships following their diagnosis and/or disease progression, as well as difficulty meeting new people since being diagnosed. Related to this, CHD symptoms (e.g., fatigue, nausea, and jaundice) were reported to impact participants’ ability to participate in social events. Participants also described taking more care around family and friends in order to prevent disease transmission.

CHD impacted physical functioning, with participants describing difficulty walking long distances, climbing stairs, exercising, and participating in sports due to fatigue and/or joint pain. Fatigue was also reported to impact participants’ stamina, requiring the need for more frequent breaks when engaging in physical activities. Impacts on daily activities were also frequently mentioned, including difficulty completing chores or running errands (e.g., carrying groceries, cleaning, cooking); such impacts were most commonly attributed to fatigue and pain. Participants also reported difficulty eating and loss of appetite due to nausea and vomiting.

Impacts on work were also reported. Participants described difficulties meeting job requirements including completing work tasks that required lifting objects and engaging with clients and colleagues, describing concerns about stigma and disease transmission. Consequently, some participants reported working reduced hours or no longer working due to their CHD symptoms.

Nearly half of participants reported experiencing stigma associated with CHD, with forms of social stigma most commonly mentioned. In particular, participants described how misconceptions related to the cause and transmission of the disease led to others making incorrect assumptions or negative comments regarding their sexual history or use of illicit drugs. Some participants also reported receiving rejections from potential sexual partners and noted the discomfort they felt with having to explain their CHD to potential partners. Social stigma was also experienced from family and friends. Participants described how on receiving their HDV diagnosis family and friends tended to treat or look at them differently or ‘turn their backs’, leaving them to feel socially isolated. Additionally, a few participants reported forms of internalized stigma, including negative self-perceptions regarding their health status compared to others and feelings of shame regarding the severity of their diagnosis.

CHD was also reported to impact sleep, with participants describing difficulty falling and/or staying asleep due to pain, nausea, or generally feeling unwell. A small number of participants also described impacts to their finances, including the cost of medical appointments and treatments and the impact of reduced working hours on their income.

### Saturation analysis

Only two new symptom concepts were identified in the final set of interviews: anemia and dizziness. These concepts were only mentioned once and by one participant each. No new impact domains were identified in the final set of interviews. Taken together, results of the saturation analysis suggest the sample size was sufficient to elicit the core signs, symptoms, and impacts of CHD and that saturation was achieved. The saturation grids are available in Supplementary File S2.

### Cognitive debriefing of the HQLQv2

#### Understanding of instructions

Participants were asked if they understood the instructions used throughout the HQLQv2. Table 4 presents an overview of instruction comprehension. Except for instruction 8, all instructions were understood by all participants who were asked.

**Table 4:** Overview of instruction Understanding (*N* = 39)

Instruction	Clearly understood, *n* (%)
**Instruction 1 – Primary Instruction**: This survey asks for your views about your health. This information will help keep track of how you feel and how well you are able to do your usual activities. For each of the following questions, please mark an X in the one box that best describes your answer.	39/39 (100%)
**Instruction 2 – Physical Functioning domain**: The following questions are about activities you might do during a typical day. Does your health now limit you in these activities? If so, how much?	39/39 (100%)
**Instruction 3 – Role-Physical domain**: During the past 4 weeks, how much of the time have you had any of the following problems with your work or regular daily activities as a result of your physical health?	39/39 (100%)
**Instruction 4 – Role-Emotional domain**: During the past 4 weeks, how much of the time have you had any of the following problems with your work or regular daily activities as a result of any emotional problems?	39/39 (100%)
**Instruction 5 – Mental Health domain**: These questions are about how you feel and how things have been with you during the past 4 weeks. For each question, please give the one answer that comes closest to the way you have been feeling. How much of the time during the past 4 weeks…	37/37 (100%)
**Instruction 6 – Vitality domain**: How TRUE or FALSE is each of the following statements for you?	35/35 (100%)
**Instruction 7 – Health Distress, Positive Wellbeing, and Hepatitis-Specific Health Distress domains**: How much of the time during the past 4 weeks…	33/33 (100%)
**Instruction 8 – Hepatitis-Specific Limitations domain**: How much of the time during the past 4 weeks has your hepatitis limited you in:	34/35 (94.1%)

#### Item Understanding

Item comprehension was assessed based on participants’ ability to demonstrate a clear understanding of the item wording, explain the item in their own words, or appropriately answer any follow-up questions regarding the item. All HQLQv2 items were understood by ≥ 94.9% of participants, with 36/51 items clearly understood by the entire sample.

### Item relevance

**Fig. 4:**
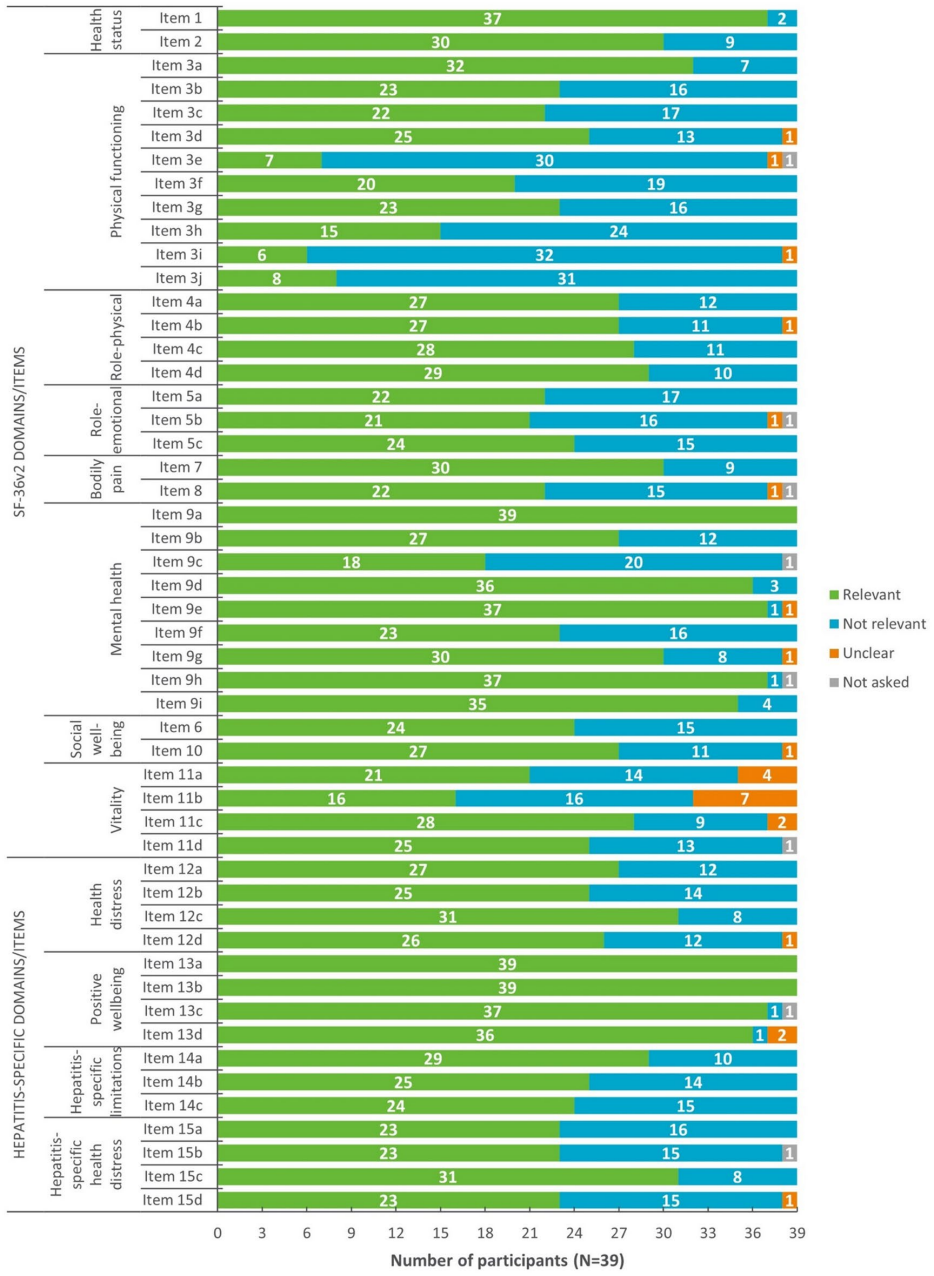
Overview of item relevance for the HQLQv2 (*N* = 39)

An overview of item relevance is presented in Fig. 4. Most items (46/51) were considered relevant to at least 50.0% of participants. Items that were relevant to less than 50.0% of participants were all from the SF-36v2 and included four items of the Physical Functioning domain (Item 3e: ‘climbing one flight of stairs’; Item 3 h: ‘walking several hundred yards’; Item 3i: ‘walking one hundred yards’; and Item 3j: ‘bathing/showering’) and one item of the Mental Health domain (Item 9c: ‘down in the dumps’). Exemplary quotes for item relevance are provided in Table 5 (note: a single item for each domain is displayed as an example).

**Table 5:** Example quotes for relevance of select HQLQv2 items to the patient experience of CHD (*N* = 39)

HQLQv2 Domain/item	Example supportive quote
**Physical functioning** Item 3a. Vigorous activities	“*For example*,* if I need to lift a table because we are currently shooting*,* I need to ask someone else to do it*,* because I simply can’t. Or if I have to do it because I have no choice*,* I’m going to sweat like hell*,* and feel really tired…*” (Participant 30)
**Role-Physical** Item 4c. Limitations to work and other activities	*“… things that I used to like very much*,* such as teaching theatre lessons*,* I gave up because the commuting*,* plus the time I spent on teaching made me super exhausted*,* and…certain ordinary activities of my work*,* they had to take away from me some tasks I used to do because I couldn’t finish them.”* (Participant 15)
**Mental health** Item 9 g. Feeling worn out	*“That’s not having any strength*,* being knackered*,* tired… and… as I said*,* a little of the times because this disease sometimes leads me to being fatigued and tired.”* (Participant 11)
**Social wellbeing** Item 10. Interference with social activities	*“…it always depends on my physical health*,* not on my emotional state. By physical health I mean*,* if I’m in pain or I’m not feeling well*,* this might interfere with my activities a little bit.”* (Participant 36)
**Health distress** Item 12b. Weighted down	*…it is not always the same because family and friend come to cheer me up… when I suffer from pains or when I feel tired*,* yes well in those cases I feel demoralized.”* (Participant 29)
**Positive wellbeing** Item 13c. Cheerful	*“…So if I was happy*,* if I was excited… yes*,* in the last four weeks… Because as I said*,* I do make a good use of the time and I try to do… good things.* (Participant 11)
**Hepatitis-specific limitations** Item 14b. Daily work limitations	*“Because*,* mostly*,* the housework… I don’t do that anymore. Because I know it’s going to affect me. Ehh*,* about my daily work*,* sometimes*,* it affects me so much that I simply don’t do it.”* (Participant 17)
**Hepatitis-specific health distress** Item 15d. Frustrated	*“…they want to know how I handle it psychologically. If I am angry about having Hepatitis. Of course I am.”* (Participant 5)

### Understanding of response options and recall periods

Most participants (*n* = 38/39; 97.4%) correctly endorsed or reported no concerns with the response options for at least 48/51 items, with 51.3% demonstrating an understanding for all items.

Participant understanding was also assessed for the three recall periods used throughout the HQLQv2. The vast majority of participants understood and appropriately considered the recall period of a ‘typical day’ and ‘one-year’ when selecting their response to the corresponding items (both: *n* = 38/39; 97.4%). For items with a recall period of the ‘past 4 weeks’, more than two-thirds of participants (*n* = 25/39; 64.1%) demonstrated an understanding for all items. The remaining 14 participants considered a timeframe greater than 4 weeks (e.g., previous year, past few months) when selecting a response to at least one item.

### Ease of use and appropriateness of the HQLQv2

Following item-level debriefing, participants were asked to provide general feedback on the HQLQv2. Most participants who were asked found the HQLQv2 clear and easy to understand (*n* = 21/24; 87.5%) and relevant to their experience of CHD (*n* = 35/37; 94.6%).

### Cognitive debriefing of the FSS

#### Understanding and relevance

All participants demonstrated a clear understanding of the FSS instructions. Item comprehension was very high, with all nine items understood by ≥ 94.9% of participants. All participants correctly endorsed or reported no concerns with the response options for at least 6/9 items, with 69.2% demonstrating an understanding for all items. In most cases, participants who did not demonstrate an understanding for at least one item selected a response option that did not match their reported experience of the concept assessed. Most participants (*n* = 32/39; 82.1%) understood and appropriately considered the recall period of ‘the past week’ when selecting a response to all items. Five participants; however, considered a longer timeframe (e.g., ‘past few months’, ‘past four weeks’) than specified for at least one item.

**Fig. 5:**
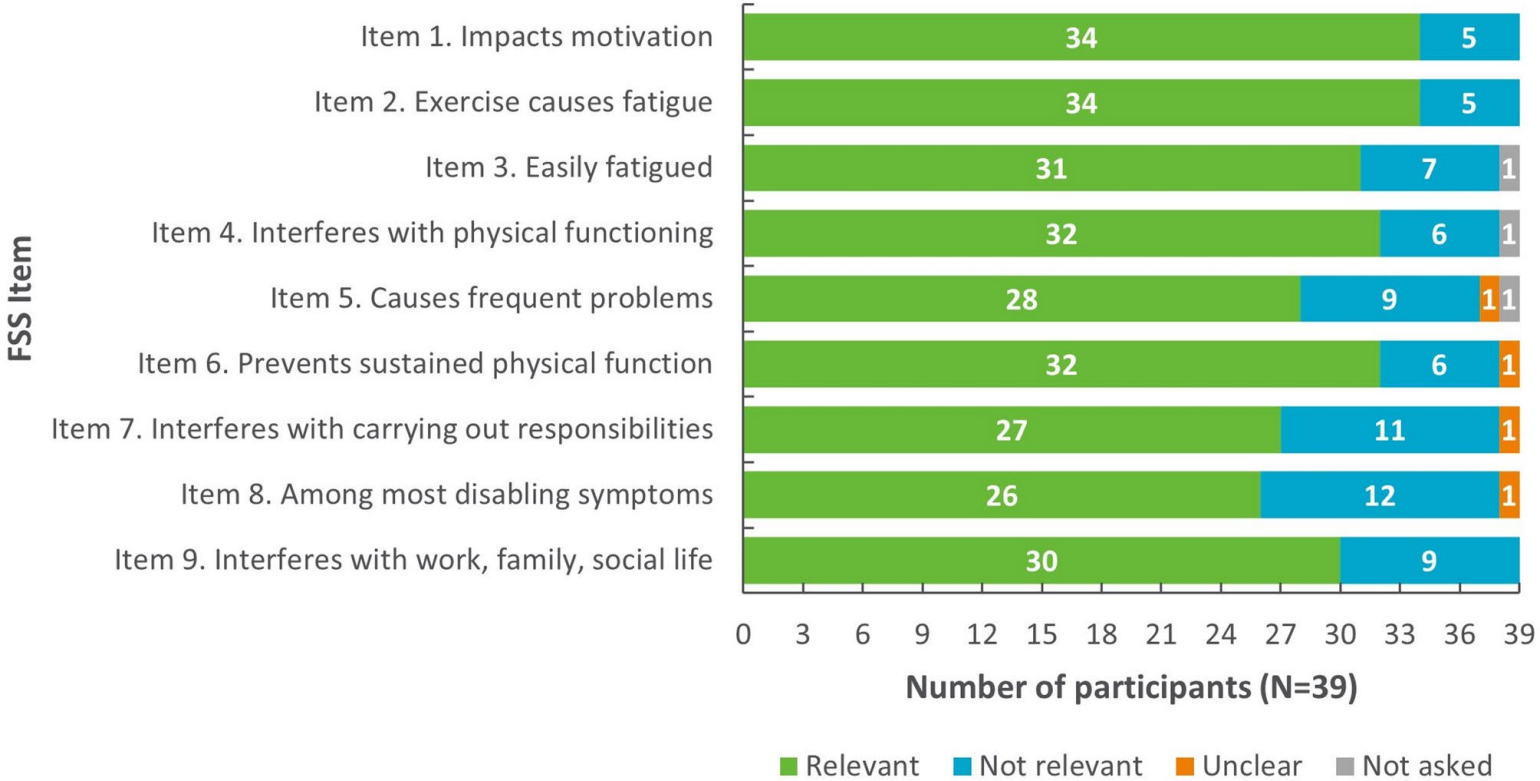
Overview of item relevance for the FSS (*N* = 39)

Participants confirmed that the items in the FSS were relevant to their experience of CHD (see Fig. 5; Table 6 for example quotes). At least 73.7% of participants endorsed 7/9 items as relevant. The remaining two items were reported to be relevant by 69.2% and 66.7% of participants, respectively.

**Table 6:** Example quotes for relevance of FSS items to the patient experience of CHD (*N* = 39)

FSS item	Example supportive quote
**Item 1**. Impacts motivation	*“Because when I am so tired*,* I have difficulty in finding the motivation to do things even the easiest and simplest one as taking care of the house… so I totally agree.”* (Participant 23)
**Item 2**. Exercise causes fatigue	*I get tired while doing exercise. I would say 6 because today physical activities are limited*,* maybe a walk very slow or normal path*,* a light swimming… I don’t practice any sports as in the past*,* like playing tennis*,* going for a run or doing the marathon.* (Participant 26)
**Item 3**. Easily fatigued	*“I am easily fatigued…. because it’s true*,* I get easily tired*,* I start working at 9:00 am and by 11:00 I’m already tired.”* (Participant 30)
**Item 4**. Interferes with physical functioning	*“Fatigue limits me absolutely. So*,* when I am fatigued then my body is fatigued as well*,* and I am not active anymore.”* (Participant 11)
**Item 5**. Causes frequent problems	*“Because when I am tired*,* I am very irritated. And when I am irritated then that causes problems within my family*,* with my husband.”* (Participant 5)
**Item 6**. Prevents sustained physical function	*“Well*,* if I was very tired*,* I couldn’t be active for a longer time*,* I couldn’t stand it for long*,* in a physical sense. I needed breaks*,* or I even had to lay down.”* (Participant 9)
**Item 7**. Interferes with responsibilities	*“Yes*,* I’d say 7*,* because as I said before*,* it doesn’t matter what my duties and obligations are*,* if I don’t rest*,* I can’t carry out any of them.”* (Participant 33)
**Item 8**. Among most disabling symptoms	*“Yes. I suffer from 2 symptoms as I told you before but fatigueness is the most limiting so I would put 7.”* (Participant 24)
**Item 9**. Interferes with work, family, social life	*“I could say 7 because I totally agree. Yes*,* I wouldn’t say 6 but 7 because I agree*,* completely agree with it. Fatigue affects my life from a professional*,* social and familial point of view.”* (Participant 29)

### Ease of use and appropriateness of the FSS

Most participants who were asked found the FSS clear and easy to understand (*n* = 21/24; 87.5%) and relevant to their experience of CHD (*n* = 31/39; 79.5%). Those participants who did not consider the FSS relevant either did not experience fatigue due to CHD (*n* = 4) or did not consider fatigue a significant burden on their daily life (*n* = 2). One participant suggested that while fatigue is associated with CHD, it is less relevant to their experience as other factors not associated with the disease may contribute to them feeling tired.

### Conceptual coverage

The HQLQv2 and FSS demonstrated strong conceptual coverage of the most frequently reported impact domains identified in the CE interviews (Table 7). Both measures include items assessing physical functioning, social functioning, activities of daily living, and work. The HQLQv2 also assesses emotional wellbeing. Neither measure includes items assessing impacts of hepatitis on sleep, stigma or finances; however, these were less frequently mentioned during the interviews. This suggests the HQLQv2 and FSS assess impact concepts most proximal to the experience of CHD, while the other impact domains not included in the measures may be considered more distal (or influenced by external factors or other impacts).

**Table 7:** Conceptual coverage of the HQLQv2 and FSS (*N* = 39)

Impact domain (*n*)*	Impacts	Hepatitis Quality of Life Questionnaire (HQLQv2)	Fatigue Severity Scale (FSS)
Emotional wellbeing (*n* = 31, 79.5%)	Anxiety and worry	✓	**X**
	Depression and low mood	✓	**X**
	Stress	✓	**X**
	Anger and annoyance	✓	**X**
	Emotional fatigue	✓	**X**
Physical functioning (*n* = 29, 74.4%)	Overall	**X**	✓
	Exercising	✓	✓
	Walking long distance	✓	**X**
	Climbing stairs	✓	**X**
Social functioning (*n* = 29, 74.4%)	Overall	**X**	✓
	Attending social events	✓	**X**
	Meeting new people	✓	**X**
Activities of daily living (*n* = 27, 69.2%)	Overall	**X**	✓
	Chores	✓	**X**
	Running errands	✓	**X**
	Hygiene activities	✓	**X**
Work (*n* = 23, 59.0%)	Overall	**X**	✓
	Meeting job responsibilities	✓	**X**
	Working reduced hours	✓	**X**
Sleep (*n* = 19, 48.7%)	Overall	**X**	**X**
	Falling asleep	**X**	**X**
	Staying asleep	**X**	**X**
Stigma (*n* = 18, 46.2%)	Overall	**X**	**X**
	Social stigma	**X**	**X**
	Internalized stigma	**X**	**X**
Finances (*n* = 6, 15.4%)	Overall	**X**	**X**
	Medical appointments	**X**	**X**

*n = number of participants who reported the impact domain during the interviews; ✓ = impact domain/concept assessed; X = impact domain/concept not assessed

Note: where ‘overall’ is used, this indicates that the measure assessed the impact domain broadly, rather than individual concepts

## Discussion

Appropriate PRO measures that assess key concepts of interest to patients are needed to evaluate treatment benefits from a patient perspective. Qualitative methods remain the gold standard for eliciting detailed patient experience data regarding the signs, symptoms, and impacts that matter most to patients [9], and are particularly well-suited for exploring a debilitating disease like CHD. To the best of our knowledge, this is the first qualitative study to comprehensively explore the experience of CHD from the patient perspective, in line with regulatory guidance [9, 10]. Findings confirm the importance of measuring HRQoL to evaluate the impact of CHD on patients’ lived experience and support focused assessment of the impact of fatigue on patients’ HRQoL. The HQLQv2 and FSS were identified as potential PROs suitable for the assessment of HRQoL in patients with CHD. However, content validity of these measures had not yet been established in a CHD population. The overall objective of this study was to generate evidence that the content of these measures effectively assesses the core impacts of chronic HDV infection, through the conduct of combined concept elicitation and cognitive debriefing interviews.

The signs, symptoms, and impacts reported in this study were used to inform the development of a conceptual model, illustrating the patient experience of CHD. The concepts identified in this research in this research were broadly consistent with those identified in scientific literature [5, 6, 8], including the recent qualitative study in patients with CHB [7]. CE findings demonstrated that the concepts assessed by the HQLQv2 and FSS were those most frequently mentioned by participants during the CE interviews (and captured in the respective conceptual model), providing evidence these measures assess the most important and relevant HRQoL impacts associated with CHD. The most frequently reported domains of impact were limitations to physical functioning (e.g., difficulty walking long distances), social functioning (e.g., difficulty attending social events), emotional wellbeing (e.g., anxiety and worry about disease transmission), ADL (e.g., difficulty completing household chores), and work (e.g., difficulty performing job role), all of which are assessed by items in the HQLQv2. These findings substantiate existing literature, highlighting the considerable impact of CHD on patients’ HRQoL [8]. Notably, participants most commonly attributed these impacts to fatigue, which they described as a severe and particularly burdensome symptom affecting nearly every aspect of their daily lives. These findings align with previous literature [36] and confirm that fatigue is a clinically relevant symptom directly associated with patients’ HRQoL, providing support for use of the FSS in this population.

Relevance and comprehension of the HQLQv2 and FSS were confirmed in CD interviews, further supporting their use as potential endpoints in CHD clinical trials to assess the effect of treatment on HRQoL. Across both measures, participants demonstrated a clear and consistent understanding of the items, instructions, response options, and recall periods. Most items assessed by HQLQv2 and FSS were also relevant to participants’ experience of CHD. Items that were relevant to less than half of participants were all from the SF-36v2 portion of the HQLQv2 and included four items assessing aspects of physical functioning (i.e., ‘climbing a single flight of stairs’, ‘walking several hundred yards, ‘walking one hundred yards’, and ‘bathing and showering’) and one item assessing mental health (i.e., ‘feeling down in the dumps’). Given the SF-36v2 is a generic measure for use across different health conditions, it could be expected that some of the concepts assessed by this measure may not be as relevant to the specific patient experience of CHD. However, these findings may also be explained by the sample, with fewer severe disease patients (i.e., F3 and F4) represented. Although no clear differences were observed in the proportion of patients who reported each of the items as relevant, it is possible that the concepts assessed by these items may be more relevant to those with more severe liver fibrosis. Notably, all items of the two hepatitis-specific domains of the HQLQv2 (i.e., hepatitis-specific limitations and health distress) were relevant to most participants (range: 59%-79%), supporting their suitability for use to assess the effect of bulevirtide on patients’ HRQoL. Insights obtained from use of these measures have value beyond traditional safety and efficacy messaging and can be used to support the generation of value propositions and provide evidence of product differentiation [12].

A strength of this research is the inclusion of patients from four geographically diverse locations in Europe (i.e., Germany, Italy, and Spain) and the US, ensuring broader applicability of the findings across countries and cultures. It should be acknowledged that only two participants were recruited from the US. While this means that US-based participants are underrepresented in the sample, this reflects the low prevalence of CHD in the US and highlights the overall rarity of the disease [37]. The interview sample also included patients with a good representation of key demographic and clinical characteristics. In particular, a range of educational levels were included, demonstrating the consistency of understanding of the PRO measures across the target population. However, fewer patients with more severe liver disease (i.e., stages F3 and F4) and those with active HCC were recruited than planned. Due to the severity of their illness, it is possible these patients were less willing or unable to participate compared to those with less severe disease. This could impact the generalizability of the findings, as fewer severe signs, symptoms, and less impacts may have been elicited in this research.

A requirement of this research was also for participants to be fluent in the principal language of the target country. While prevalence of HDV in Europe is increasing, this has largely been driven by immigrant populations coming from regions endemic for the virus [38]. As many of these individuals may have not met the language proficiency requirements for the study, their experience of CHD may not be reflected in this research. Additionally, as HDV requires co-infection with HBV for its replication, participants may have had difficulty attributing their signs, symptoms, and associated impacts to CHD only. Such difficulty has been previously described in the literature [39]. Despite this, the findings reported in this study could be considered to reflect the patient experience of both CHB and CHD infection together. Further, while a conceptual model should normally be developed initially based on a review of available published literature [9], no qualitative studies detailing the patient experience of CHD were identified in the existing published research. Saturation analysis, however, provided evidence the sample size was sufficient for eliciting the core signs/symptoms and impacts of CHD, with only two new symptom concepts identified in the final set of interviews. While anemia is a common finding in patients with liver disease [40], symptoms of anemia (e.g., fatigue and weakness) are more likely to be reported by patients and is reflective of the results of this research. No studies were identified that listed dizziness as a key symptom of viral hepatitis.

## Conclusion

This study makes an important contribution to the literature by providing valuable qualitative insights into the patient experience of CHD. The findings supported the development of a conceptual model providing a comprehensive depiction of the patient experience and provided evidence supporting the content validity of the HQLQv2 and FSS as outcome measures suitable for use in patients with CHD.

## Electronic supplementary material

Below is the link to the electronic supplementary material.

Supplementary Material 1

Supplementary Material 2

## Data Availability

The datasets generated and/or analyzed during the current study are not publicly available to protect participant confidentiality.

## Abbreviations

ADL: Activities of Daily Living
CD: Cognitive Debriefing
CE: Concept Elicitation
CHB: Chronic Hepatitis B
CHD: Chronic Hepatitis D
FDA: Food and Drug Administration
FSS: Fatigue Severity Scale
HBV: Hepatitis B Virus
HCC: Hepatocellular Carcinoma
HCV: Hepatitis C Virus
HE: Hepatic Encephalopathy
HIV: Human Immunodeficiency Virus
HRQoL: Health-Related Quality of Life
HQLQv2: Hepatitis Quality of Life Questionnaire version 2
IFNα: Interferon alpha
IRB: International Review Board
ISPOR: International Society of Pharmacoeconomics and Outcomes Research
PFDD: Patient-Focused Drug Development
PRO: Patient-Reported Outcome
RNA: Ribonucleic Acid
SF-36: Short Form-36
US: United States
WCG: Western Copernicus Group

## Declarations

### Competing of interest

Pietro Lampertico is a paid speaker and/or advisor for Roche Pharma/Diagnostics, Gilead Sciences,GSK, AbbVie, Janssen, MYR, Eiger, Antios, Aligos, VIR, Grifols, Altona, and Roboscreen. Aishwarya Chohan and Rowena Jones are employees of Adelphi Values Ltd, a health outcomes agency, who were paid by the study sponsor (Gilead Sciences Inc.) to conduct the research described in this manuscript and to write this manuscript. Hannah Elwick was an employee of Adelphi Values Ltd at the time of research and is now employed by the University of Nottingham. Nicola Williamson was an employee of Adelphi Values Ltd at the time of research and is now employed by UCB. Caroline Burk and Marvin Rock are employees of Gilead Sciences Inc, the study sponsor. Alon Yehoshua was an employee of Gilead Sciences Inc at the time of research. Robert Gish is a paid consultant and/or advisor toAbbott, AbbVie, Altimunne, Antios, Arrowhead, Dynavax, Eiger, Eisai, Enyo, Genentech, Genlantis, Gerson Lehrman Group, Gilead Sciences, Helios, HepaTX, HepQuant, Intercept, Janssen, Merck, Pfizer, Topography Health, Venatorx and Prodigy, holds grants from Gilead, and holds stock options in Eiger, Genlantis, HepQuant AngioCrine, HepaTx. Nancy Reau is a paid consultant for Gilead, VIR,AbbVie, Abbott, and Salix and holds grants with Eiger, AbbVie, Gilead, and Salix. Heiner Wedemeyer is a paid consultant and/or speaker for Aligos, Altimmune, Biotest, BMS, BTG, Dicerna, Enanta, Gilead,Janssen, Merck/MSD, Roche and VIR, holds grants from Biotest, Gilead, Merck/MSD, Roche and isinvolved in clinical trials for Altimmune, BMS, Gilead, Janssen, Merck/MSD, Novartis and VIR. Maria Buti is a paid consultant for Gilead.
