# Supplementary material for: Exploring the patient experience of chronic hepatitis D (CHD) and assessment of content validity of the Hepatitis Quality of Life Questionnaire and (HQLQv2) and the Fatigue Severity Scale (FSS)
Source: J Patient Rep Outcomes. 2025 Jul 7;9:84. doi: 10.1186/s41687-025-00903-3 (PMC12234919; doi:10.1186/s41687-025-00903-3)
Supplement: Supplementary file 1 — Supplementary Material 1 [file 41687_2025_903_MOESM1_ESM.docx]

# 1 Interview objectives and overview

The overall objectives of this study are to understand the experience of patients having chronic Hepatitis Delta Virus (HDV) infection and to cognitively debrief two patient reported outcomes (PRO), the Hepatitis Quality of Life Questionnaire (HQLQ) and the Fatigue Severity Scale (FSS) to evaluate the content validity and appropriateness of these PRO measures for use in HDV. This will be achieved by the conduct of combined qualitative concept elicitation (CE) and cognitive debriefing (CD) interviews with individuals with HDV.

## Interview objectives

The specific aims of the combined CE and CD interviews are:

- To identify, explore and characterize key concepts related to signs/symptoms, the impact on daily activities and health-related quality of life and the overall burden of HDV, from the perspectives of individuals living with the condition.
- To evaluate the content validity of the HQLQv2 and FSS by assessing understanding, relevance and comprehension of the instructions and concepts assessed in the PRO measures, suitability of the response options and recall periods, and by identifying any missing concepts.

## Overview of the interview process

The interview should be conducted as follows:

1. **Part 1**: Introductions (5 minutes).
2. **Part 2**: Concept elicitation discussion: Experience of HDV (20 minutes).
3. **Part 3**: Cognitive debriefing of the HQLQv2 and FSS (35 minutes).

In total, the interview should take approximately **60 minutes.** Halfway through the interview (i.e., between Part 2 and Part 3), please check if the participant needs to take a break, particularly if the participant becomes tired.

## Materials

To conduct this interview **you, the interviewer, will need**:

- The interview guide in electronic or paper form (this document).
- Audio-recording device or recording capabilities on Microsoft Teams/WebEx.
- Pen/pencil and paper or tracking table to take notes during the interview.

# Instructions to Interviewer

## The role of the interviewer before the interview

- Review the relevant documents prior to the interview: Check that Adelphi Values have copies of the participant’s signed Informed Consent Form, completed demographic form, and completed Case Report Form (CRF). All documentation should be thoroughly checked for completeness prior to beginning the interview. Finally, check that the participant has copies of the PRO measures to hand, as these will be debriefed during the interview. These PRO measures should have been sent to the participant in advance of the interview. It is important that the participant does not look at these PRO measures until instructed to do so during the interview.

## The role of the interviewer during the interview

- **Digital audio recording**: Ensure that participant’s voice throughout the interview is clearly recorded by the audio-recording. The audio recording will be transcribed verbatim. If using an audio-recorder, check the volume settings and positioning to ensure the clarity of the audio-recording prior to the start of the interview. Check that the audio-recorder has enough battery power for the duration of the interview. Avoid rustling papers near the recorder or jostling the recorder during the interview as this will lead to inaudible responses.
- **Interview guide:** Use this guide as a guide not a script – feel free to use your own wording in the framing of questions where relevant and follow the lead of the participant in terms of phrasing and terminology and the order of questions (i.e., when a participant raises an issue that comes up later in the guide, it is fine to address the issue earlier and not to bring it up again later). **This will require in-depth knowledge of the guide**. Where possible, please ensure that each question in this interview guide is asked.
- **Nonverbal communication (applicable for video-call interviews only):** Make a written note in of any non-verbal communication by the participant (e.g., vocalizations or facial expressions indicating rejection or acceptance of one of your comments; evidence of confusion, frustration, annoyance, etc.). Comment on the non-verbal cue and invite the participant to explain their feelings to ensure you have properly interpreted the gesture.

The role of the interviewer is to **inquire** and **support:**

- Do not give your own point of view;
- Be patient and accept silence to make sure the participant has time to think about his/her response;
- Make sure you are non-judgemental in your tone when probing and make clear to the participant that it is okay if they do not know what a question means or if they are experiencing problems reading the question;
- Help the participant stick to the topics intended in the guide (but not necessarily in the order presented in the guide);
- Help the participant to avoid repeating themselves by knowing the guide and following up on topics when raised;
- Help the participant cross-reference and make connections that they would not do spontaneously;
- Ask the participant to clarify any comment or explain a vague reference that is pertinent to the research question (e.g., if they talk about “it” and “that”, ask them what is meant by these terms).
- If the participant directs any questions to you that are of a medical nature, please explain that you are not a medical professional and that they should direct any medical queries to their doctor or healthcare provider.
- Hepatitis D is a stigmatized condition, and therefore some discussions may be sensitive and/or upsetting. If the participant becomes upset during the interview, offer them a break and remind them they can stop the interview at any point, or postpone the interview to another day. If the participant wishes to continue the interview, ask if they would be comfortable discussing the current question, or if they would like to move on to another topic.

## Avoid biasing or leading the participant

As much as possible your questions should be open-ended and general, rather than specific. You should avoid closed questions, easily answered ‘yes’ or ‘no’. It is especially important that you do not lead the participant with your questions or remarks that may bias their responses. You should avoid using statements or questions that lead the participant or encourage social or treatment desirability or agreement bias in response to the wording or framing of the questions you pose. Allow the participant enough time to answer. Encourage participants to explain his or her responses to ensure a clear understanding of the intent of the participant.

Below most questions in both the CE and CD sections of this guide we have provided a number of possible probes that should help the participant explain their thinking. These probes are **optional** and do not have to be asked if the participant spontaneously addresses the topics noted in the probes. Ideally, probes will not be asked as they could potentially bias the participant’s response – the probes are only provided for use when the participant finds it difficult to answer your questions, their response requires clarification, or where the participant does not provide a sufficient amount of in-depth information in their spontaneous response.

You do not need to ask the questions/probes exactly as they are written in this guide. There may be instances where you need to invent your own probes or reword the probes specified in the interview guide so as to be relevant to the participant’s responses and the interview situation. Feel free to phrase a question differently so long as you do so without leading the participant or deviating from the intention of the original question. Remember that this is just a guide and you do not need to ask the questions in exactly the way they are written.

POSSIBLE NON-LEADING PROBES MIGHT INCLUDE:

- Can you describe exactly what that means for you?
- Tell me more about that.
- How does that affect you?
- Can you talk more about ___________?
- What do you think are the reasons for that?
- How often might that happen?

During cognitive debriefing, it is important for you to follow the questions in this guide as closely as possible, but it may still be necessary for you to reword your questions in order to aid the participant’s understanding (DO NOT REWORD THE ITEMS). Please follow the interview guide carefully during this section, ensuring that for each item being debriefed in the CD section of the interview you know whether the:

- Item was correctly interpreted and **understood** by the participant.
- Item measured a concept that was **relevant** to the participant.
- Item would/would not be **reworded** by the participant.
- Item **response options** were appropriate and well understood by the participant.

If the participant directs any questions to you that are of a medical nature, please explain that you are not a medical professional and that the participant should direct any medical queries to their doctor or nurse.

## Using the interview guide

Throughout this guide we have formatted the instructions and questions so that it is clear which instructions are for the interviewer’s reference, and which instructions and questions are intended for the participant, and which of these are compulsory questions and which are probes (to be used when necessary). The formatting is as follows:

| **UPPER CASE TEXT** | These are guidance notes/instructions for you (the interviewer). These are in bold blue text. |
| --- | --- |
|  | These are statements and instructions that you read aloud to the participant. |
| 1. **Bold text** | Questions prefixed by a specific number and in bold text must be posed to all participants |
| - *Italic text* | All probes or follow up questions are bulleted – these do not necessarily need to be used but are included to assist you in drawing further information from participants if needed. |

# Adverse event reporting

An adverse event (AE) is:

***‘Any untoward medical occurrence when a patient administered a sponsor medicinal product, and which does not necessarily have to have a causal relationship with this treatment. An adverse event can be any unfavourable and unintended sign (e.g., an abnormal laboratory finding), symptom, or disease temporally associated with the use of a medicinal product, whether or not considered related to the medicinal product.’***

Remember, an AE must be reported when the following are present:

**PREP – Patient, Reporter, Event, Product**

1. **A Patient or group of patients**

Identifiers such as age, age group, birth date, gender, role, profession should be collected if available, if not the event should still be reported.

1. **An identifiable Reporting source**

Information that identifies the reporter, establishing knowledge of the reportable event in an identifiable consumer. The reporter could be a patient, doctor, patient’s parent, friend, colleague or caregiver.

1. **An adverse Event**

Description of at least one event or product complaint.

1. **A suspect Product**

Patient must mention taking one of the products being marketed by the company that we are doing research for.

**Remember:** The event need not have a causal relationship with the treatment or usage.

An AE can also include a complaint specific to the product itself, or packaging, as opposed to its effect on the patient. Examples include damaged or missing tablets; wrong strength or colour of tablets; damaged packaging; a label that cannot be read; a liquid that should be clear but is cloudy or contains unexpected particles; a bent needle, a broken syringe; a missing patient information leaflet or the identification of a potentially counterfeit medicine.

A **serious adverse event** (which Gilead must report to the relevant local and global regulatory authorities) is any undesirable experience associated with the use of a medical product in a patient when the patient outcome is:

- **Death**

Report if you suspect that the death was an outcome of the adverse event and include the date if known.

- **Life-threatening**

Report if suspected that the patient was at substantial risk of dying at the time of the adverse event or use or continued use of the device or other medical product might have resulted in the death of the patient.

- **Hospitalization (initial or prolonged)**

Report if admission to the hospital or prolongation of hospitalization was a result of the adverse event.

Emergency room visits that do not result in admission to the hospital should be evaluated for one of the other serious outcomes (E.g., life-threatening; required intervention to prevent permanent impairment or damage; other serious medically important event).

- **Disability or permanent damage**

Report if the adverse event resulted in a substantial disruption of a person's ability to conduct normal life functions, i.e., the adverse event resulted in a significant, persistent or permanent change, impairment, damage or disruption in the patient's body function/structure, physical activities and/or quality of life.

- **Congenital anomaly/birth defect**

Report if you suspect that exposure to a medical product prior to conception or during pregnancy may have resulted in an adverse outcome in the child.

- **Required intervention to prevent permanent impairment or damage (devices)**

Report if you believe that medical or surgical intervention was necessary to preclude permanent impairment of a body function, or prevent permanent damage to a body structure, either situation suspected to be due to the use of a medical product.

- **Other serious (important medical events)**

Report when the event does not fit the other outcomes, but the event may jeopardize the patient and may require medical or surgical intervention (treatment) to prevent one of the other outcomes. Examples include allergic bronchospasm (a serious problem with breathing) requiring treatment in an emergency room, serious blood dyscrasias (blood disorders) or seizures/convulsions that do not result in hospitalization. The development of drug dependence or drug abuse would also be examples of important medical events.

**Special Situation Reports (SSRs)**

Gilead also requires other safety information to be reported in Special Situation Reports (SSR) regardless of whether associated with and AE or not. SSRs must include the following information:

- Pregnancy (maternal pregnancy and partner pregnancy);
- Exposure via breast feeding;
- Occupational exposure;
- Unexpected benefits;
- Counterfeit or falsified medicine;
- Abuse/misuse;
- Lack of effect;
- Overdose;
- Off label use;
- Transmission of infectious agents via a product;
- Drug interactions;
- Medication error.

**All safety information must be reported to Gilead, even if:**

- The AE or SSR is already known and included in the product’s package insert;
- The patient has said that they have already sent it to Gilead or a regulatory authority;
- The patient recovered from the AE or SSR.

At the time an AE/SSR in mentioned during the interview please say the following to the patient:

- “During the interview you mentioned a problem that you experienced while taking [product name] a Gilead product. Gilead, the sponsor of this research needs to collect information about their products to continue to make them as safe and effective as possible. Every report they receive contains potentially useful information. I would like to spend a couple of minutes with you now to collect some more details about the problems. Is that OK with you?”
- **If yes:** “Thank you. The information you provide will be sent to the study sponsor, who may wish to contact you and your doctor for further information. If you agree to provide your contact details, we will share them with the sponsor. This will not be linked in any way to the other things we have talked about during this interview. Are you happy to provide your contact details?”
- **If no:** “OK that’s fine. However, as the information you were given about this study explained, we do have to report this problem to the sponsor. This will be done anonymously, and none of your personal details will be shared with them.”

You should complete the **‘AE Form’** (**Error! Reference source not found.**) with as much detail as you can retrieve.

You must report any AEs or SSRs to Gilead (via email to Safety_FC@gilead.com) using the **‘AE Form’** (**Error! Reference source not found.**) for all Gilead products, even for Gilead products not in scope of the research, within one business day of being informed about it. You will receive an Acknowledgement of Receipt for any safety reports submitted to Gilead including a Gilead Reference Number (MCN). You must retain the MCN for use during the reconciliation process. Please refer to the protocol for further information about how to report AEs.

# Part 1: Interview introduction and consent (5 minutes)

## Introductions

**INTRODUCE YOUSELF AS WORKING FOR ADELPHI VALUES**

- Thank you for taking the time to talk to us today.
- My name is [first name]. I work for a company called Adelphi Values. Adelphi Values is a health outcomes research company that works with pharmaceutical companies to better understand the experience of health conditions such as HDV.

**EXPLAIN THE AIMS AND PROCESS OF THE INTERVIEW**

- The aim of this research is to learn more about the experience of individuals with Hepatitis Delta virus (HDV) and how the condition affects their lives.
- In the first part of the interview, I am going to ask you some questions about your experience of living with HDV, specifically the symptoms you experience and how it affects your daily life.
- In the second part of the interview, I will ask you to complete and give feedback on two questionnaires that ask about your experience of HDV.
- This interview should last approximately 60 minutes. If you need to take a break at any point, please just let me know.

**REASSURE THE PARTICIPANT OF CONFIDENTIALITY AND ANONYMITY**

- Anything you tell me during the interview will be kept confidential. Any information about you, and the responses you provide, will be given a unique ID number and will only be accessible to the project team who are conducting this study.
- A word for word written version of the interview may be shared with the sponsor, but any information that may identify you, like names or places will be removed, and you will be assigned an ID number so that the sponsor cannot identify you.
- This study is being paid for by a company who make medicines. If you report a bad experience or problem that you have experienced because of taking a medication made by the study sponsor, I will need to let them know about this. Any information you provide will be treated in confidence and used solely for the purpose of drug safety monitoring. If you do not want to give me your contact details, I will still let the sponsor know about the problem you had, but I will do this anonymously (so it cannot be linked back to you), and none of your personal details will be shared with them.

## Audio-recording the interview

- The interview today will be audio-recorded to allow me to pay careful attention to what you say and to make certain we accurately capture what you tell me during the interview. Please try to speak loudly and clearly so that your comments can be heard on the audio-recording.
- Please be honest in your responses and don’t be afraid to voice any opinions, there are no right or wrong answers to these questions.

## Verbal consent

**TURN ON THE AUDIO-RECORDER**

- I will now switch on the audio-recorder.
- This is [name of interviewer] with participant [participant ID number] on [date of interview] at [time of interview]. The interview is being conducted by [telephone/Microsoft Teams]. The interview is being conducted in [state location of interview including city and country e.g., Manchester, UK].
- The participant [participant ID number] is based in [state location including city and country e.g., Chicago, USA].
- Do you agree to take part in the interview?
- Do you agree to have this interview audio-recorded?
  - **[IF PARTICIPANT DOES NOT AGREE TO HAVE THE INTERVIEW RECORDED: Thank the participant for taking part and end the interview and switch off the recorder.]**
- Do you have any questions before we start?
- OK, I’ll start now, OK?

# Part 2: Concept elicitation: Exploration of experience of Hepatitis Delta Virus (20 minutes)

## Experience of Hepatitis Delta Virus (10 minutes)

- In the first section of the interview, I will ask you general questions about your experience of having Hepatitis Delta Virus, what symptoms you experience and how they affect you in your daily life, if at all.

**ASK THE PARTICIPANT HOW THEY REFER TO THEIR CONDITION AND USE THIS TERMINOLOGY THROUGHOUT THE INTERVIEW.**

1. **When you talk to your family or friends about your condition, how do you refer to it? What do you call it?**

- OK, so for the rest of the interview, I will use [participant term for HDV] if that’s OK?

1. **Thinking about your HDV, please can you tell me about what first led you to talk to your doctor (or seek medical advice)?**
2. **Please tell me about your current experience of [participant term for HDV].**
   - *What is it like to live with [participant term for HDV]?*
3. **Tell me about the symptoms that you experience as part of [participant term for HDV].**

**NOTE EVERY SYMPTOM AND IMPACT THAT THE PARTICIPANT MENTIONS SPONTANEOUSLY IN THE SYMPTOM TRACKER IN** [**APPENDIX B**](#_Appendix_B:_Symptom) **AND THE IMPACT TRACKER IN** [**APPENDIX C**](#_Appendix_C:_Impacts)**. FOR EACH SYMPTOM IN** [**APPENDIX B**](#_Appendix_B:_Symptom) **(BOTH SPONTANEOUS AND PROBED) ASK THE PARTICIPANT THE FOLLOWING QUESTIONS AND MAKE A NOTE OF THEIR RESPONSES IN** [**APPENDIX B**](#_Appendix_B:_Symptom)**.**

**FOR EACH IMPACT IN** [**APPENDIX C**](#_Appendix_C:_Impacts) **(BOTH SPONTANEOUS AND PROBED) ASK THE PARTICIPANT THE FOLLOWING QUESTIONS AND MAKE A NOTE OF THEIR RESPONSES IN** [**APPENDIX C**](#_Appendix_C:_Impacts)**.**

| For each symptom mentioned, ask the following questions: | |
| --- | --- |
| **Symptom description** | - **How would you describe this symptom?** - **Tell me about any additional words you use to describe this symptom?** |
| **Onset** | - **When did you first experience this symptom?** |
| **Location** | - **Where in the body do you experience this symptom?** |
| **Frequency** | - **How often do you experience this symptom?** - Daily/Weekly/monthly/other? - **How does the frequency of this symptom vary? Please tell me about that.** - How does the frequency of this symptom vary during a flare-up? Please tell me about that. - How has the frequency of this symptom changed over time? Please tell me about that. |
| **Severity** | - **How would you rate this symptom on a typical day on a scale of 0-10, where 0 is not at all severe and 10 is the worst you can imagine? Can you tell me why you chose that rating?** - How does the severity of this symptom vary? Please tell me about that. - How does the severity of this symptom vary during a flare-up? Tell me about that. |
| **Duration** | - **How long does this symptom typically last for?** - How does the duration of this symptom vary? - How has the duration of this symptom changed over time? Please tell me about that. |
| **Timing** | - **When do you experience this symptom during the day?** - **How does this symptom vary during the day if at all?** - **Do you experience this symptom at night at all? Please tell me about that.** |
| **Flare ups** | - **Please tell me about any flare ups that you experience of this symptom.** - How does this symptom change when you experience a flare up? |
| **Attribution** | - **What do you think is the cause of this symptom?** - Do you think this symptom is due to Hepatitis B/[participant term for HDV]/Other? - **Is there anything that makes this symptom worse?** - **Is there anything that makes this symptom better?** |
| **Impact on daily life**  **[MAKE A NOTE OF RESPONSES IN IMPACT TRACKER IN** [**APPENDIX C**](#_Appendix_C:_Impacts)**]** | - **How does this symptom affect your daily life? Please tell me about that.** - Are there things you are not able to do or that are more difficult because of this symptom? - Is there anything you avoid doing when you have this symptom? - How often do you experience this impact? - How long does this impact last? - How severe would you say this impact is? |

1. **Do you experience any other symptoms of [participant term for HDV] that we have not yet talked about?**
   - **IF YES:** *Please tell me more about that.*

- I have written down the different symptoms you just discussed. They are [**READ ALOUD ALL OF THE SYMPTOMS THE PARTICIPANT HAS REPORTED**].

1. **Which of these symptoms are most bothersome to you? Why?**
2. **If there was a new treatment to improve the symptoms of [participant term for HDV], which of these symptoms would be most important to treat and why?**
   - *How would the symptom have to change?*
     - *E.g., Frequency, severity, duration, level of impact.*

## Impacts of Hepatitis Delta Virus (10 minutes)

- We are now going to talk about what it’s like to live with [participant term for HDV] and the ways in which it may affect different aspects of your life.

**PARTICIPANTS MAY HAVE ALREADY MENTIONED A NUMBER OF IMPACTS SPONTANEOUSLY WHEN DISCUSSING THEIR SYMPTOMS. PROBE ON THESE IMPACTS FIRST, USING THE PROBES FROM THE RELEVANT IMPACT DOMAIN SECTIONS BELOW (QUESTIONS 6-13).**

1. **You mentioned [impact] earlier, please can you tell me more about that?**

**AFTER YOU HAVE PROBED ON ALL SPONTANEOUSLY REPORTED IMPACTS, ENCOURAGE SPONTANEOUS REPORTING OF ANY OTHER IMPACTS NOT PREVIOUSLY MENTIONED. NOTE ANY SPONTANEOUSLY MENTIONED IMPACTS IN THE IMPACT TRACKING TABLE (**[**APPENDIX C**](#_Appendix_C:_Impacts)**). USE THE PROBES FROM THE RELEVANT IMPACT DOMAIN SECTIONS BELOW FOR ANY IMPACTS SPONTANEOUSLY MENTIONED HERE.**

1. **Can you tell me about any other ways in which your life is affected by [participant term for HDV]?**

### Physical functioning

1. **In what ways, if at all, does [participant term for HDV] affect you physically?**
   - *What physical activities are affected? Please tell me about that.*

**[TO BE ASKED FOR EACH ACTIVITY MENTIONED]**

- - *How is the activity affected? (e.g., do you stop doing it, does it make it more difficult, do you take longer to do it)?*

### Activities of daily living

1. **In what ways, if at all, does [participant term for HDV] affect your daily activities?**
   - *What daily activities are affected? Please tell me about that.*
     - *E.g., housework/chores, washing, getting dressed, hobbies*

**[TO BE ASKED FOR EACH ACTIVITY MENTIONED]**

- - *How is the daily activity affected? (e.g., do you stop doing it, does it make it more difficult, do you take longer to do it)*

### Sleep

1. **In what ways, if at all, does [participant term for HDV] affect your sleep?**
   - *How does [*participant term for HDV*] affect your sleep? Tell me about that.*
     - *E.g., difficulty falling asleep, how much sleep you get in a night, waking during the night, struggling to get back to sleep.*

### Emotional well-being

1. **In what ways, if at all, does [participant term for HDV] affect you emotionally?**
   - *How does having [participant term for HDV] make you feel?*

### Social well-being

1. **In what ways, if at all, does [participant term for HDV] affect you socially (e.g., your ability to socialize or take part in social activities)?**

- *What social activities are affected? Please tell me about that.*
  - - *E.g., going out with friends, spending time with family, participating in social activities, going to restaurants/bars)*

1. **In what ways, if at all, does [participant term for HDV] affect your relationships? Please tell me about that.**

### Stigma

1. **In what ways, if at all, do you experience stigma due to your [participant term for HDV]?**
   - *What does stigma mean to you?*
   - *Can you provide examples of how stigma affects you due to your [participant term for HDV]?*
   - *Can you describe when others first treated you differently because of your [participant term for HDV]?*
   - *In what ways, if at all, do others treat you differently because of your [participant term for HDV]?*
   - *How do you cope when people treat you differently? Do you do anything differently?*

### Work

1. **In what ways, if at all, does [participant term for HDV] affect your ability to work?**
   - *How does [participant term for HDV] affect your productivity, if at all?*

*How does [participant term for HDV] affect how quickly you work? Tell me about this.*

- - *Do you have to take time off work/sick leave?*

### Finances

1. **In what ways, if at all, does [participant term for HDV] affect you financially?**
   - *E.g., cost of medication, reduced income due to time off work.*
2. **What aspect of [participant term for HDV] affects your life the most? Why?**
3. **Are there any other ways [participant term for HDV] impacts you that we have not discussed that you think are important to talk about?**

# Part 3: Cognitive debriefing of the FSS and HQLQ (35 minutes)

**ENSURE THE PARTICIPANT HAS A PAPER OR DIGITAL COPY OF THE HEPATITIS QUALITY OF LIFE QUESTIONNAIRE (HQLQ;** [**APPENDIX D**](#_Appendix_D:_Hepatitis)**), AND THE Fatigue Severity Scale (FSS;** [**APPENDIX E**](#_Appendix_E:_EQ-5D-5XL)**) IN FRONT OF THEM FOR THIS SECTION OF THE INTERVIEW**

**INTRODUCE THE PRO MEASURES TO THE PARTICIPANT:**

- We are now going to talk through the Hepatitis Quality of Life Questionnaire (HQLQv2) and the Fatigue Severity Scale (FSS).
- Please read aloud each instruction and each question, one by one. For each question, please say which answer you would choose and why. Please tell me any thoughts or opinions you have as you read them.
- If anything you read or are instructed to do is unclear please let me know. If you feel that anything is unsuitable or irrelevant to your experience of [participant term for HDV], please say it as it occurs to you.
- After you answer each question, I am going to ask you some additional questions about your understanding of the question and whether you think it is relevant to your experience of [participant term for HDV]. I will also ask you about the response option you selected and what it would mean to you to select a different response option.
- Please be honest in your responses and do not be afraid to voice any of your opinions. There are no right or wrong answers and if there are things you do not like or find difficult to understand, we want to know about it so we can improve the questionnaire for others.
- We want you to say any thoughts or opinions that come to mind while answering the questions. I understand that this is not the way people are typically used to sharing their thoughts, so I might remind you to ‘think-aloud’.
- The questions that I ask you might be a bit repetitive, but they are important, so we can see how you respond to each question and what your thoughts and opinions are on each.
- Do you have any questions at this point?
- Please start by reading the instructions, speaking your thoughts aloud as you go.

**MAKE A NOTE OF THE RESPONSE GIVEN, WHETHER THE PARTICIPANT UNDERSTOOD THE INSTRUCTION/ITEM.**

## Hepatitis Quality of Life Questionnaire version 2 (HQLQv2)

| Table 1. Cognitive debriefing of the HQLQv2 | | | |
| --- | --- | --- | --- |
| UNDERSTANDING | RELEVANCE | RESPONSE OPTION | RECALL PERIOD |
| **[INSTRUCTION 1] - This survey asks for your views about your health. This information will help keep track of how you feel and how well you are able to do your usual activities. For each of the following questions, please mark an ⌧ in the one box that best describes your answer.** | | | |
| - ***In your own words what is this instruction asking you to do?*** |  |  |  |
| **[ITEM 1] - In general, would you say your health is:** | | | |
| - ***In your own words, what is this question asking you?*** | - *What answer would you choose for this question? Why?* |  | - *What time period were you thinking of when you answered this question?* |
| **[ITEM 2] - Compared to one year ago, how would you rate your health in general now?** | | | |
| - ***In your own words, what is this question asking you to do?*** | - *What answer would you choose for this question? Why?* | - *What does ‘much better’ mean to you?* | - *What time period were you thinking of when you answered this question?* |
| **[INSTRUCTIONS Q3] - The following questions are about activities you might do during a typical day. Does your health now limit you in these activities? If so, how much?** | | | |
| - ***In your own words, what is this instruction asking you?*** |  |  |  |
| **[ITEM 3a] - Vigorous activities, such as running, lifting heavy objects, participating in strenuous sports** | | | |
| - ***In your own words, what is this question asking you?*** | - *What answer would you choose for this question? Why?* | - *What does ‘limited a little’ mean to you?* |  |
| **[ITEM 3b] - Moderate activities, such as moving a table, pushing a vacuum cleaner, bowling, or playing golf** | | | |
| - ***In your own words, what is this question asking you?*** | - *What answer would you choose for this question? Why?* | - *What does ‘limited a lot’ mean to you?* |  |
| **[ITEM 3c] - Lifting or carrying groceries** | | | |
| - ***In your own words, what is this question asking you?*** | - *What answer would you choose for this question? Why?* | - *What does ‘not limited at all’ mean to you?* |  |
| **[ITEM 3d] - Climbing several flights of stairs** | | | |
| - ***In your own words, what is this question asking you?*** | - *What answer would you choose for this question? Why?* | - *What does ‘limited a little’ mean to you?* |  |
| **[ITEM 3e] - Climbing one flight of stairs** | | | |
| - ***In your own words, what is this question asking you?*** | - *What answer would you choose for this question? Why?* | - *What does ‘limited a lot’ mean to you?* |  |
| **[ITEM 3f] - Bending, kneeling, or stooping** | | | |
| - ***In your own words, what is this question asking you?*** | - *What answer would you choose for this question? Why?* | - *What does ‘not limited at all’ mean to you?* |  |
| **[ITEM 3g] - Walking more than a mile** | | | |
| - ***In your own words, what is this question asking you?*** | - *What answer would you choose for this question? Why?* | - *What does ‘limited a little’ mean to you?* |  |
| **[ITEM 3h] - Walking several hundred yards** | | | |
| - ***In your own words, what is this question asking you?*** | - *What answer would you choose for this question? Why?* | - *What does ‘limited a lot’ mean to you?* |  |
| **[ITEM 3i] - Walking one hundred yards** | | | |
| - ***In your own words, what is this question asking you?*** | - *What answer would you choose for this question? Why?* | - *What does ‘not limited at all’ mean to you?* |  |
| **[ITEM 3j] - Bathing or dressing yourself** | | | |
| - ***In your own words, what is this question asking you?*** | - *What answer would you choose for this question? Why?* | - *What does ‘limited a little’ mean to you?* |  |
| **[INSTRUCTIONS 4] - During the past 4 weeks, how much of the time have you had any of the following problems with your work or other regular daily activities as a result of your physical health?** | | | |
| - ***In your own words, what is this instruction asking you?*** |  |  | - *What time period is this instruction asking you to think about?* |
| **[ITEM 4a] - Cut down on the amount of time you spent on work or other activities** | | | |
| - ***In your own words, what is this question asking you?*** | - *What answer would you choose for this question? Why?* | - *What does ‘a little of the time’ mean to you?* |  |
| **[ITEM 4b] - Accomplished less than you would like** | | | |
| - ***In your own words, what is this question asking you?*** | - *What answer would you choose for this question? Why?* | - *What does ‘none of the time’ mean to you?* |  |
| **[ITEM 4c] - Were limited in the kind of work or other activities** | | | |
| - ***In your own words, what is this question asking you?*** | - *What answer would you choose for this question? Why?* | - *What does ‘all of the time’ mean to you?* |  |
| **[ITEM 4d] - Had difficulty performing the work or other activities (for example, it took extra effort)** | | | |
| - ***In your own words, what is this question asking you?*** | - *What answer would you choose for this question? Why?* | - *What does ‘most of the time’ mean to you?* |  |
| **[INSTRUCTIONS 5] - During the past 4 weeks, how much of the time have you had any of the following problems with your work or other regular daily activities as a result of any emotional problems (such as feeling depressed or anxious)?** | | | |
| - ***In your own words, what is this instruction asking you?*** |  |  | - *What time period is this instruction asking you to think about?* |
| **[ITEM 5a] - Cut down on the amount of time you spent on work or other activities** | | | |
| - ***In your own words, what is this question asking you?*** | - *What answer would you choose for this question? Why?* | - *What does ‘some of the time’ mean to you?* |  |
| **[ITEM 5b] - Accomplished less than you would like** | | | |
| - ***In your own words, what is this question asking you?*** | - *What answer would you choose for this question? Why?* | - *What does ‘a little of the time’ mean to you?* |  |
| **[ITEM 5c] - Did work or other activities less carefully than usual** | | | |
| - ***In your own words, what is this question asking you?*** | - *What answer would you choose for this question? Why?* | - *What does ‘none of the time’ mean to you?* |  |
| **[ITEM 6] - During the past 4 weeks, to what extent has your physical health or emotional problems interfered with your normal social activities with family, friends, neighbors, or groups?** | | | |
| - ***In your own words, what is this question asking you?*** | - *What answer would you choose for this question? Why?* | - *What does ‘slightly’ mean to you?* | - *What time period were you thinking of when you answered this question?* |
| **[ITEM 7] - How much bodily pain have you had during the past 4 weeks?** | | | |
| - ***In your own words, what is this question asking you?*** | - *What answer would you choose for this question? Why?* | - *What does ‘severe’ mean to you?* | - *What time period were you thinking of when you answered this question?* |
| **[ITEM 8] - During the past 4 weeks, how much did pain interfere with your normal work (including both work outside the home and housework)?** | | | |
| - ***In your own words, what is this question asking you?*** | - *What answer would you choose for this question? Why?* | - *What does ‘extremely’ mean to you?* | - *What time period were you thinking of when you answered this question?* |
| **[INSTRUCTION 9] - These questions are about how you feel and how things have been with you during the past 4 weeks. For each question, please give the one answer that comes closest to the way you have been feeling. How much of the time during the past 4 weeks…** | | | |
| - ***In your own words, what is this instruction asking you?*** |  |  | - *What time period is this instruction asking you to think about?* |
| **[ITEM 9a] - Did you feel full of life?** | | | |
| - ***In your own words, what is this question asking you?*** | - *What answer would you choose for this question? Why?* | - *What does ‘none of the time’ mean to you?* | - *What time period were you thinking of when you answered this question?* |
| **[ITEM 9b] - Have you been very nervous?** | | | |
| - ***In your own words, what is this question asking you?*** | - *What answer would you choose for this question? Why?* | - *What does ‘most of the time’ mean to you?* |  |
| **[ITEM 9c] - Have you felt so down in the dumps that nothing could cheer you up?** | | | |
| - ***In your own words, what is this question asking you?*** | - *What answer would you choose for this question? Why?* | - *What does ‘some of the time’ mean to you?* |  |
| **[ITEM 9d] - Have you felt calm and peaceful?** | | | |
| - ***In your own words, what is this question asking you?*** | - *What answer would you choose for this question? Why?* | - *What does ‘a little of the time’ mean to you?* |  |
| **[ITEM 9e] - Did you have a lot of energy?** | | | |
| - ***In your own words, what is this question asking you?*** | - *What answer would you choose for this question? Why?* | - *What does ‘none of the time’ mean to you?* |  |
| **[ITEM 9f] - Have you felt downhearted and depressed?** | | | |
| - ***In your own words, what is this question asking you?*** | - *What answer would you choose for this question? Why?* | - *What does ‘all of the time’ mean to you?* |  |
| **[ITEM 9g] - Did you feel worn out?** | | | |
| - ***In your own words, what is this question asking you?*** | - *What answer would you choose for this question? Why?* | - *What does ‘some of the time’ mean to you?* |  |
| **[ITEM 9h] - Have you been happy?** | | | |
| - ***In your own words, what is this question asking you?*** | - *What answer would you choose for this question? Why?* | - *What does ‘most of the time’ mean to you?* |  |
| **[ITEM 9i] - Did you feel tired?** | | | |
| - ***In your own words, what is this question asking you?*** | - *What answer would you choose for this question? Why?* | - *What does ‘a little of the time’ mean to you?* |  |
| **[ITEM 10] - During the past 4 weeks, how much of the time has your physical health or emotional problems interfered with your social activities (like visiting with friends, relatives, etc.)?** | | | |
| - ***In your own words, what is this question asking you?*** | - *What answer would you choose for this question? Why?* | - *What does ‘none of the time’ mean to you?* | - *What time period were you thinking of when you answered this question?* |
| **[INSTRUCTION 11] - How TRUE or FALSE is each of the following statements for you?** | | | |
| - *In your own words, what is this instruction asking you to do?* |  |  |  |
| **[ITEM 11a] - I seem to get sick a little easier than other people** | | | |
| - ***In your own words, what is this question asking you?*** | - *What answer would you choose for this question? Why?* | - *What does ‘mostly true’ mean to you?* |  |
| **[ITEM 11b] - I am as healthy as anybody I know** | | | |
| - ***In your own words, what is this question asking you?*** | - *What answer would you choose for this question? Why?* | - *What does ‘mostly false’ mean to you?* |  |
| **[ITEM 11c] - I expect my health to get worse** | | | |
| - ***In your own words, what is this question asking you?*** | - *What answer would you choose for this question? Why?* | - *What does ‘definitely true’ mean to you?* |  |
| **[ITEM 11d] - My health is excellent** | | | |
| - ***In your own words, what is this question asking you to do?*** | - *What answer would you choose for this question? Why?* | - *What does ‘definitely false’ mean to you?* | - *What time period were you thinking of when you answered these questions?* |
| **[INSTRUCTION 12] - How much of the time during the past 4 weeks...** | | | |
| **[ITEM 12a] - Were you discouraged by your health problems?** | | | |
| - ***In your own words, what is this question asking you?*** | - *What answer would you choose for this question? Why?* | - *What does ‘all of the time’ mean to you?* | - *What time period were you thinking of when you answered this question?* |
| **[ITEM 12b] - Did you feel weighted down by your health problems?** | | | |
| - ***In your own words, what is this question asking you?*** | - *What answer would you choose for this question? Why?* | - *What does ‘a good bit of the time’ mean to you?* |  |
| **[ITEM 12c] - Was your health a worry in your life?** | | | |
| - ***In your own words, what is this question asking you?*** | - *What answer would you choose for this question? Why?* | - *What does ‘a little of the time’ mean to you?* |  |
| **[ITEM 12d] - Were you frustrated by your health?** | | | |
| - ***In your own words, what is this question asking you?*** | - *What answer would you choose for this question? Why?* | - *What does ‘none of the time’ mean to you?* |  |
| **[INSTRUCTION 13] - How much of the time during the past 4 weeks...** | | | |
| **[ITEM 13a] - Have you generally enjoyed the things you do?** | | | |
| - ***In your own words, what is this question asking you?*** | - *What answer would you choose for this question? Why?* | - *What does ‘all of the time’ mean to you?* | - *What time period were you thinking of when you answered this question?* |
| **[ITEM 13b] - Has your daily life been full of things that were interesting to you?** | | | |
| - ***In your own words, what is this question asking you?*** | - *What answer would you choose for this question? Why?* | - *What does ‘most of the time’ mean to you?* |  |
| **[ITEM 13c] - Have you felt cheerful, lighthearted?** | | | |
| - ***In your own words, what is this question asking you?*** | - *What answer would you choose for this question? Why?* | - *What does ‘a good bit of the time’ mean to you?* |  |
| **[ITEM 13d] - Has living been a wonderful adventure for you?** | | | |
| - ***In your own words, what is this question asking you?*** | - *What answer would you choose for this question? Why?* | - *What does ‘a little of the time’ mean to you?* |  |
| **[INSTRUCTION 14] - How much of the time during the past 4 weeks has your hepatitis limited you in:** | | | |
| - ***In your own words, what is this instruction asking you?*** |  |  | - *What time period is this instruction asking you to think about?* |
| **[ITEM 14a] - Your everyday physical activities such as walking or climbing stairs, carrying groceries or participating in sports?** | | | |
| - ***In your own words, what is this question asking you?*** | - *What answer would you choose for this question? Why?* | - *What does ‘none of the time’ mean to you?* | - *What time period were you thinking of when you answered this question?* |
| **[ITEM 14b] - Your daily work, both work outside the home and housework?** | | | |
| - ***In your own words, what is this question asking you?*** | - *What answer would you choose for this question? Why?* | - *What does ‘most of the time’ mean to you?* |  |
| **[ITEM 14c] - Your normal social activities with family, friends, neighbors or groups?** | | | |
| - ***In your own words, what is this question asking you?*** | - *What answer would you choose for this question? Why?* | - *What does ‘a good bit of the time’ mean to you?* |  |
| **[INSTRUCTION 15] - How much of the time during the past 4 weeks...** | | | |
| **[ITEM 15a] - Were you discouraged because of your hepatitis?** | | | |
| - ***In your own words, what is this question asking you to do?*** | - *What answer would you choose for this question? Why?* | - *What does ‘a little of the time’ mean to you?* | - *What time period were you thinking of when you answered this question?* |
| **[ITEM 15b] - Did you feel weighted down by your hepatitis?** | | | |
| - ***In your own words, what is this question asking you to do?*** | - *What answer would you choose for this question? Why?* | - *What does ‘none of the time’ mean to you?* |  |
| **[ITEM 15c] - Was having hepatitis a worry in your life?** | | | |
| - ***In your own words, what is this question asking you?*** | - *What answer would you choose for this question? Why?* | - *What does ‘all of the time’ mean to you?* |  |
| **[ITEM 15d] - Were you frustrated because of having hepatitis?** | | | |
| - ***In your own words, what is this question asking you?*** | - *What answer would you choose for this question? Why?* | - *What does ‘most of the time’ mean to you?* |  |

## General feedback on the HQLQv2

- We would also like to hear any general feedback you have about the questionnaire you have just looked at

1. **What do you think of this questionnaire in general?**
   - *What did you like/dislike about it?*
2. **How easy or difficult was it to answer the questions?**
   - *Were there any questions that were particularly difficult to answer or not relevant to your experience? Tell me about those.*
3. **Is there anything that you would change about the questionnaire? What?**
   - *Are there any questions that should be added or removed when thinking about the most important symptoms and impacts of [participant term for HDV] that you experience?*
4. **How relevant do you think this questionnaire is to your experience with [participant term for HDV]? Tell me about that.**
5. **Do you have any further comments about the questionnaire that we haven’t already discussed?**

## Fatigue Severity Scale (FSS)

| Table 2. Cognitive debriefing of the FSS | | |
| --- | --- | --- |
| UNDERSTANDING | RELEVANCE | RESPONSE OPTION |
| **[INSTRUCTION 1] Below are a series of statements regarding your fatigue. By fatigue we mean a sense of tiredness, lack of energy or total body give-out. Please read each statement and choose a number from 1 to 7, where # 1 indicates you completely disagree with the statement and # 7 indicates you completely agree. Please answer these questions as they apply to the past TWO WEEKS.** | | |
| - ***In your own words what is this instruction asking you to do?*** |  |  |
| **[ITEM 1] My motivation is lower when I am fatigued.** | | |
| - ***In your own words, what is this question asking you?*** | - *What answer would you choose for this question? Why?* | - *How would you indicate your chosen response?* - *What would ‘3’ mean on this item?* |
| **[ITEM 2] Exercise brings on my fatigue.** | | |
| - ***In your own words, what is this question asking you?*** | - *What answer would you choose for this question? Why?* | - *How would you indicate your chosen response?* - *What would ‘1’ mean on this item?* |
| **[ITEM 3] I am easily fatigued.** | | |
| - ***In your own words, what is this question asking you?*** | - *What answer would you choose for this question? Why?* | - *How would you indicate your chosen response?* - *What would ‘7’ mean on this item?* |
| **[ITEM 4] Fatigue interferes with my physical functioning.** | | |
| - ***In your own words, what is this question asking you?*** | - *What answer would you choose for this question? Why?* | - *How would you indicate your chosen response?* - *What would ‘5’ mean on this item?* - *What is the difference between a score of ‘5’ and a score of ‘7’?* |
| **[ITEM 5] Fatigue causes frequent problems for me.** | | |
| - ***In your own words, what is this question asking you?*** | - *What answer would you choose for this question? Why?* | - *How would you indicate your chosen response?* - *What would ‘2’ mean on this item?* |
| **[ITEM 6] My fatigue prevents sustained physical functioning.** | | |
| - ***In your own words, what is this question asking you?*** | - *What answer would you choose for this question? Why?* | - *How would you indicate your chosen response?* - *What would ‘4’ mean on this item?* |
| **[ITEM 7] Fatigue interferes with carrying out certain duties and responsibilities.** | | |
| - ***In your own words, what is this question asking you?*** | - *What answer would you choose for this question? Why?* | - *How would you indicate your chosen response?* - *What would ‘2’ mean on this item?* |
| **[ITEM 8] Fatigue is among my most disabling symptoms.** | | |
| - ***In your own words, what is this question asking you?*** | - What answer would you choose for this question? Why? | - *How would you indicate your chosen response?* - *What would ‘6’ mean on this item?* |
| **[ITEM 9] Fatigue interferes with my work, family or social life.** | | |
| - ***In your own words, what is this question asking you?*** | - *What answer would you choose for this question? Why?* | - *How would you indicate your chosen response?* - *What is the difference between a score of ‘1’ and a score of ‘5’?* |

## General feedback on the FSS

- We would also like to hear any general feedback you have about the questionnaire you have just looked at

1. **What do you think of this questionnaire in general?**
   - *What did you like/dislike about it?*
2. **How easy or difficult was it to answer the questions?**
   - *Were there any questions that were particularly difficult to answer or not relevant to your experience? Tell me about those.*
3. **What time period were you thinking of when you were answering the questions?**
   - *Was it difficult or easy to think about this time period?*
4. **Is there anything that you would change about the questionnaire? What?**
   - *Are there any questions that should be added or removed when thinking about the most important symptoms and impacts of [participant term for HDV] that you experience?*
5. **How relevant do you think this questionnaire is to your experience with [participant term with HDV]? Tell me about that.**
6. **Do you have any further comments about your experience with [participant term for HDV] or the questionnaires that you have read today that we haven’t already discussed?**

## End of interview

**TURN OFF THE AUDIO-RECORDER**

- **This is the end of the interview, thank you very much for your participation. Please contact your doctor if you have any concerns about your condition or treatment.**
