# Supplementary material for: Exploring the patient experience of chronic hepatitis D (CHD) and assessment of content validity of the Hepatitis Quality of Life Questionnaire and (HQLQv2) and the Fatigue Severity Scale (FSS)
Source: J Patient Rep Outcomes. 2025 Jul 7;9:84. doi: 10.1186/s41687-025-00903-3 (PMC12234919; doi:10.1186/s41687-025-00903-3)
Supplement: Supplementary file 2 — Supplementary Material 2 [file 41687_2025_903_MOESM2_ESM.docx]

| Supplementary Table 1. Conceptual saturation of symptom concepts | | | | | | | | | | | | | | | | | | | | | | | | | | | | | | | | | | | | | | | |
| --- | --- | --- | --- | --- | --- | --- | --- | --- | --- | --- | --- | --- | --- | --- | --- | --- | --- | --- | --- | --- | --- | --- | --- | --- | --- | --- | --- | --- | --- | --- | --- | --- | --- | --- | --- | --- | --- | --- | --- |
| Symptoms | Group 1 (n=10) | | | | | | | | | | Group 2 (n=10) | | | | | | | | | | Group 3 (n=10) | | | | | | | | | | Group 4 (n=9) | | | | | | | | |
|  | IT 01 | IT 02 | IT 03 | DE01 | DE 03 | DE 04 | DE 05 | DE 06 | DE 07 | DE 08 | DE 09 | DE 10 | ES 05 | ES 06 | ES07 | ES08 | ES09 | ES10 | ES11 | ES14 | ES 16 | US 02 | IT 05 | IT 06 | IT 07 | IT 08 | IT 09 | IT 10 | IT 11 | ES 17 | US 03 | ES 18 | ES19 | IT 13 | IT 14 | IT 15 | DE 11 | DE 12 | DE 13 |
| Fatigue | **🗸** | **🗸** | **🗸** | **🗸** | **🗸** | **🗸** |  | **🗸** | **🗸** | **🗸** | **🗸** | **🗸** | **🗸** |  | **🗸** | **🗸** |  | **🗸** |  | **🗸** | **🗸** |  | **🗸** | **🗸** | **🗸** | **🗸** | **🗸** | **🗸** | **🗸** | **🗸** |  |  | **🗸** | **🗸** | **🗸** | **🗸** | **🗸** | **🗸** | **🗸** |
| Nausea |  |  | **🗸** | **🗸** | **🗸** |  | **🗸** |  |  |  |  | **🗸** | **🗸** |  |  | **🗸** |  |  |  | **🗸** |  |  | **🗸** | **🗸** |  | **🗸** |  |  | **🗸** |  |  |  | **🗸** | **🗸** | **🗸** | **🗸** |  |  | **🗸** |
| Joint pain |  |  | **🗸** |  |  |  |  | **🗸** |  |  |  |  | **🗸** |  | **🗸** | **🗸** |  | **🗸** |  | **🗸** |  |  | **🗸** |  |  | **🗸** |  | **🗸** | **🗸** | **🗸** |  |  |  |  |  |  |  | **🗸** | **🗸** |
| Loss of appetite |  | **🗸** | **🗸** | **🗸** |  |  |  |  |  |  |  |  | **🗸** |  | **🗸** |  |  | **🗸** |  | **🗸** | **🗸** |  | **🗸** | **🗸** |  | **🗸** |  |  |  |  |  |  | **🗸** |  | **🗸** |  |  |  |  |
| Pain over liver |  |  | **🗸** |  |  |  | **🗸** |  | **🗸** |  | **🗸** |  |  |  | **🗸** | **🗸** |  | **🗸** | **🗸** | **🗸** |  |  |  |  |  |  |  |  |  |  |  | **🗸** | **🗸** |  |  | **🗸** |  |  |  |
| Fever | **🗸** |  |  |  |  |  |  | **🗸** |  |  |  |  | **🗸** | **🗸** |  | **🗸** |  |  |  |  |  |  |  |  |  |  |  |  |  | **🗸** |  |  |  | **🗸** |  |  |  |  |  |
| Vomiting |  |  | **🗸** |  | **🗸** |  |  |  |  |  |  |  |  |  | **🗸** | **🗸** |  |  |  | **🗸** |  |  |  |  |  |  |  |  |  |  |  |  |  |  |  |  |  |  |  |
| Pale colored stools |  |  |  |  |  |  |  |  |  |  | **🗸** |  | **🗸** |  | **🗸** |  |  |  |  |  | **🗸** |  |  |  |  |  |  |  |  |  |  | **🗸** |  |  |  |  |  |  |  |
| Dark urine |  |  |  |  |  |  |  |  |  |  |  |  |  |  |  |  |  |  |  | **🗸** |  |  |  |  |  |  |  |  |  |  |  | **🗸** | **🗸** |  |  | **🗸** |  |  | **🗸** |
| Jaundice |  |  |  |  |  |  |  |  |  |  | **🗸** |  |  |  |  |  |  |  |  |  |  |  |  |  |  |  |  |  |  |  |  |  | **🗸** |  |  | **🗸** |  |  |  |
| Abdominal pain |  |  | **🗸** |  |  |  | **🗸** |  |  |  |  |  |  |  | **🗸** |  |  |  |  |  |  |  |  |  |  |  |  |  | **🗸** |  |  |  |  |  |  |  |  |  |  |
| Itching |  |  |  |  | **🗸** |  |  | **🗸** | **🗸** |  |  |  |  |  |  |  |  |  |  |  |  |  |  |  |  |  |  |  |  |  |  |  |  |  |  |  |  | **🗸** |  |
| Insomnia |  |  |  |  |  |  | **🗸** |  | **🗸** |  |  |  |  |  |  |  |  | **🗸** |  |  |  |  |  |  |  | **🗸** |  |  |  |  |  |  |  |  |  |  |  |  |  |
| Neurocognitive problems |  |  |  |  |  |  |  | **🗸** |  |  |  | **🗸** |  |  | **🗸** |  |  | **🗸** |  |  |  |  |  |  |  |  |  |  |  |  |  |  |  |  |  |  |  |  |  |
| Acid reflux |  |  |  |  |  |  |  |  |  |  |  |  | **🗸** |  |  |  |  |  |  |  |  |  |  |  |  |  |  |  |  |  |  |  |  |  |  |  |  |  |  |
| Bloating |  |  |  |  |  |  |  |  |  |  |  |  | **🗸** |  | **🗸** |  |  |  |  |  |  |  |  |  |  |  |  |  |  |  |  |  |  |  |  |  |  |  | **🗸** |
| Diarrhea |  |  |  |  |  |  | **🗸** |  |  |  |  |  |  |  | **🗸** |  |  |  |  |  |  |  |  |  |  |  |  |  | **🗸** |  |  |  |  |  |  |  |  |  |  |
| Flatulence |  |  |  |  |  | **🗸** |  | **🗸** |  |  |  |  |  |  | **🗸** |  |  |  |  |  |  |  |  |  |  |  |  |  |  |  |  |  |  |  |  |  |  |  |  |
| Weakness |  |  |  |  |  |  |  |  |  |  |  |  |  | **🗸** |  |  |  |  |  |  |  |  |  | **🗸** |  | **🗸** |  |  |  |  |  |  |  |  |  |  |  |  |  |
| Ankle swelling | **🗸** |  |  |  |  |  |  |  |  |  |  |  |  |  |  |  |  |  |  |  | **🗸** |  |  |  |  |  |  |  |  |  |  |  |  |  |  |  |  |  |  |
| Migraines |  |  |  |  |  |  |  |  |  |  |  |  |  |  |  |  |  |  |  |  |  |  |  |  | **🗸** | **🗸** |  |  |  |  |  |  |  |  |  |  |  |  |  |
| Muscle pain | **🗸** |  |  |  |  |  |  |  |  |  |  |  |  |  |  |  |  |  |  |  |  |  |  |  |  |  |  |  |  |  |  |  |  |  |  | **🗸** |  |  |  |
| Blood in urine |  |  |  |  |  |  |  |  |  |  |  |  | **🗸** |  |  |  |  |  |  |  |  |  |  |  |  |  |  |  |  |  |  |  |  |  |  |  |  |  |  |
| Circulation problems |  |  |  |  |  | **🗸** |  |  |  |  |  |  |  |  |  |  |  |  |  |  |  |  |  |  |  |  |  |  |  |  |  |  |  |  |  |  |  |  |  |
| Constipation |  |  |  |  |  |  |  |  |  |  |  |  |  |  | **🗸** |  |  |  |  |  |  |  |  |  |  |  |  |  |  |  |  |  |  |  |  |  |  |  |  |
| Dizziness |  |  |  |  |  |  |  |  |  |  |  |  |  |  |  |  |  |  |  |  |  |  |  |  |  |  |  |  |  |  |  |  |  |  |  |  |  | **🗸** |  |
| Fluid retention |  |  |  |  |  |  |  |  |  |  |  |  |  |  |  |  |  |  |  |  | **🗸** |  |  |  |  |  |  |  |  |  |  |  |  |  |  |  |  |  |  |
| Hot flashes |  |  |  |  |  |  |  |  |  |  |  |  |  |  |  |  |  |  |  |  |  |  |  |  |  |  |  |  |  | **🗸** |  |  |  |  |  |  |  |  |  |
| Low blood pressure |  |  |  |  |  |  |  |  |  |  |  |  |  |  |  |  |  |  |  |  | **🗸** |  |  |  |  |  |  |  |  |  |  |  |  |  |  |  |  |  |  |
| Spots on skin |  |  |  |  | **🗸** |  |  |  |  |  |  |  |  |  |  |  |  |  |  |  |  |  |  |  |  |  |  |  |  |  |  |  |  |  |  |  |  |  |  |
| Smell sensitivity |  |  |  |  |  |  |  |  |  |  |  |  | **🗸** |  |  |  |  |  |  |  |  |  |  |  |  |  |  |  |  |  |  |  |  |  |  |  |  |  |  |
| Weight loss |  |  |  |  |  |  |  |  |  |  |  |  |  |  |  |  |  |  |  |  | **🗸** |  |  |  |  |  |  |  |  |  |  |  |  |  |  |  |  |  |  |
| *Note:* 🗸 = concept reported spontaneously; 🗸 = concept reported after interview probe. First spontaneous mention of the concept is highlighted in yellow.  *Abbreviations:* IT = Italy, ES = Spain, DE = Germany, US = United States. Number beside the abbreviation represents the participant ID number. | | | | | | | | | | | | | | | | | | | | | | | | | | | | | | | | | | | | | | | |

| Supplementary Table 2. Conceptual saturation of impact domains | | | | | | | | | | | | | | | | | | | | | | | | | | | | | | | | | | | | | | | |
| --- | --- | --- | --- | --- | --- | --- | --- | --- | --- | --- | --- | --- | --- | --- | --- | --- | --- | --- | --- | --- | --- | --- | --- | --- | --- | --- | --- | --- | --- | --- | --- | --- | --- | --- | --- | --- | --- | --- | --- |
| Domains | Group 1 (n=10) | | | | | | | | | | Group 2 (n=10) | | | | | | | | | | Group 3 (n=10) | | | | | | | | | | Group 4 (n=9) | | | | | | | | |
|  | IT 01 | IT 02 | IT 03 | DE01 | DE 03 | DE 04 | DE 05 | DE 06 | DE 07 | DE 08 | DE 09 | DE 10 | ES 05 | ES 06 | ES07 | ES08 | ES09 | ES10 | ES11 | ES14 | ES 16 | US 02 | IT 05 | IT 06 | IT 07 | IT 08 | IT 09 | IT 10 | IT 11 | ES 17 | US 03 | ES 18 | ES19 | IT 13 | IT 14 | IT 15 | DE 11 | DE 12 | DE 13 |
| Emotional wellbeing |  | **🗸** |  | **🗸** | **🗸** | **🗸** | **🗸** | **🗸** | **🗸** |  | **🗸** |  | **🗸** | **🗸** | **🗸** | **🗸** |  | **🗸** | **🗸** | **🗸** | **🗸** | **🗸** | **🗸** | **🗸** |  | **🗸** | **🗸** | **🗸** | **🗸** | **🗸** | **🗸** | **🗸** | **🗸** | **🗸** |  |  | **🗸** | **🗸** | **🗸** |
| Physical functioning | **🗸** | **🗸** | **🗸** | **🗸** | **🗸** | **🗸** | **🗸** | **🗸** | **🗸** | **🗸** |  |  | **🗸** |  | **🗸** | **🗸** |  |  | **🗸** | **🗸** | **🗸** | **🗸** | **🗸** | **🗸** |  | **🗸** | **🗸** | **🗸** | **🗸** | **🗸** |  |  | **🗸** |  |  | **🗸** | **🗸** | **🗸** | **🗸** |
| Social functioning | **🗸** | **🗸** | **🗸** |  |  | **🗸** |  |  | **🗸** |  | **🗸** | **🗸** | **🗸** |  | **🗸** | **🗸** |  | **🗸** |  | **🗸** |  |  | **🗸** | **🗸** | **🗸** | **🗸** | **🗸** | **🗸** | **🗸** | **🗸** | **🗸** |  |  | **🗸** |  | **🗸** | **🗸** | **🗸** | **🗸** |
| Work | **🗸** | **🗸** | **🗸** |  |  | **🗸** |  |  | **🗸** |  |  | **🗸** | **🗸** |  | **🗸** | **🗸** |  | **🗸** |  | **🗸** |  | **🗸** |  | **🗸** | **🗸** |  | **🗸** |  |  | **🗸** |  |  | **🗸** | **🗸** | **🗸** | **🗸** | **🗸** | **🗸** | **🗸** |
| Activities of daily living | **🗸** | **🗸** | **🗸** | **🗸** | **🗸** | **🗸** | **🗸** | **🗸** | **🗸** |  |  |  |  |  | **🗸** |  |  | **🗸** |  | **🗸** |  | **🗸** | **🗸** | **🗸** | **🗸** | **🗸** | **🗸** | **🗸** | **🗸** | **🗸** |  |  | **🗸** | **🗸** |  | **🗸** | **🗸** | **🗸** | **🗸** |
| Sleep |  | **🗸** |  | **🗸** | **🗸** |  | **🗸** | **🗸** | **🗸** | **🗸** | **🗸** | **🗸** |  |  |  | **🗸** |  | **🗸** |  | **🗸** |  |  |  |  |  | **🗸** |  |  |  | **🗸** |  |  | **🗸** |  | **🗸** |  | **🗸** | **🗸** | **🗸** |
| Stigma | **🗸** |  | **🗸** |  | **🗸** |  | **🗸** |  |  |  |  | **🗸** | **🗸** |  | **🗸** | **🗸** |  | **🗸** |  |  |  |  |  |  | **🗸** |  |  |  |  |  |  | **🗸** | **🗸** | **🗸** | **🗸** |  |  | **🗸** | **🗸** |
| Financial |  |  |  |  |  |  |  |  |  |  |  |  |  |  |  |  |  |  |  |  |  | **🗸** |  |  |  |  | **🗸** |  |  |  |  |  |  | **🗸** | **🗸** |  |  | **🗸** | **🗸** |
| *Note:* 🗸 = concept reported spontaneously; 🗸 = concept reported after interview probe. First spontaneous mention of the concept is highlighted in yellow.  *Abbreviations:* IT = Italy, ES = Spain, DE = Germany, US = United States. Number beside the abbreviation represents the participant ID number. | | | | | | | | | | | | | | | | | | | | | | | | | | | | | | | | | | | | | | | |
